# Bacterial matrix metalloproteases and serine proteases contribute to the extra-host inactivation of enteroviruses in lake water

**DOI:** 10.1038/s41396-022-01246-3

**Published:** 2022-05-11

**Authors:** Marie-Hélène Corre, Virginie Bachmann, Tamar Kohn

**Affiliations:** 1 Laboratory of Environmental Chemistry, Environmental Engineering Institute (IIE), School of Architecture, Civil and Environmental Engineering (ENAC), Ecole Polytechnique Fédérale de Lausanne (EPFL), Lausanne, Switzerland; 2 Present Address: Metabolic Modelling Group, Nestlé Institute of Health Sciences, EPFL Innovation Park Building G, 1015 Lausanne, Switzerland

**Keywords:** Water microbiology, Population dynamics, Microbial ecology

## Abstract

Enteroviruses are ubiquitous contaminants of surface waters, yet their fate in presence of microbial congeners is poorly understood. In this work, we investigated the inactivation of Echovirus-11 (E11) and Coxsackievirus-A9 (CVA9) by bacteria isolated from Lake Geneva. Incubation of E11 or CVA9 in biologically active lake water caused inactivation of 2- and 4-log_10_, respectively, within 48 h. To evaluate the antiviral action of individual bacterial species, we isolated 136 bacterial strains belonging to 31 genera from Lake Geneva. The majority of isolates (92) induced decay of at least 1.5-log_10_ of CVA9, whereas only 13 isolates induced a comparable inactivation on E11. The most extensive viral decay was induced by bacterial isolates producing matrix metalloproteases (MMPs). Correspondingly, the addition of a specific MMP inhibitor to lake water reduced the extent of inactivation for both viruses. A lesser, though significant protective effect was also observed with inhibitors of chymotrypsin-like or trypsin-like proteases, suggesting involvement of serine proteases in enterovirus inactivation in natural systems. Overall, we demonstrate the direct effect of bacterial proteases on the inactivation of enteroviruses and identify MMPs as effective controls on enteroviruses’ environmental persistence.

## Introduction

The transmission of enteric viruses by the waterborne route was first discovered in the context of poliovirus [1, 2], but has since been recognized as a continuing risk to public health. While poliovirus has been eradicated, other enteroviruses continue to challenge microbial water quality [3]. Waterborne virus transmission is highly dependent on the ability of enteroviruses to persist in the environment in response to natural stressors, yet the factors controlling environmental persistence are not fully understood. Most research to date has focused on the antiviral effects of abiotic stressors, such as sunlight or temperature [4, 5], whereas biological processes resulting in virus decay have received much less scrutiny. A better understanding of the mechanisms that shape enteroviruses population in natural communities, and especially the role of surrounding microorganisms, remains an important issue to address.

Bacteria and heterotrophic protists are the most studied microorganisms for their role as regulators of aquatic microbial food webs, and they have been found to both positively and negatively regulate enterovirus populations in freshwater environments [6–8]. Among protists, direct contact of the heterotrophic nanoflagellate *Paraphysomonas* sp. or the flagellate protozoan *Caecitellus parvulus* [7] with human Echovirus-11 (E11) was found to cause virus decay, whereas the free-living amoebae *Vermamoeba vermiformis* enhanced viral persistence by acting as an environmental reservoir for infectious Coxsackievirus-B5 [8]. Unlike protists, bacteria cannot uptake viruses, yet they may exert antiviral or protective effects via the extensive array of extracellular molecules they secrete. Some bacterial compounds such as peptidoglycan and lipopolysaccharides have been shown to protect enteric viruses through capsid stabilization [9], whereas extracellular proteolytic enzymes contribute to capsid destruction [10, 11]. A study on the inactivation of Coxsackievirus-A9 (CVA9) showed that a protease mixture (pronase) produced by *Streptomyces griseus* led to the digestion of the CVA9 capsid and the subsequent release of viral RNA [12]. As the first line of defense of enteroviruses against environmental stressors, the capsid protein matrix thus represents a virus’ “Achilles’ heel” against extracellular proteases in freshwater environments.

Proteolytic enzymes are divided into seven classes according to their catalytic type, including serine proteases, cysteine proteases, aspartic proteases, glutamic proteases, asparagine proteases, threonine proteases, and matrix metalloproteases (MMPs) [13, 14]. The most described proteases among heterotrophic bacteria found in aqueous environments belong to serine proteases and MMPs groups. While the different protease classes have not been specifically investigated with respect to their effect on enteroviruses, one study showed that CVA9 was inactivated to varying extents by different proteases, including several proteases sharing the same catalytic class [10]. Furthermore, not all enteroviruses are affected by proteases to the same extent. For example, pronase was shown to inactivate CVA7, CVA9, and CVB2 serotypes, while CVB1 and CVB3 serotypes, as well as poliovirus, were not affected [10].

The aim of this work was to determine the effect of extracellular proteolytic enzymes produced by lake water bacteria on virus persistence and to disentangle the antiviral action of different protease classes. Using water from Lake Geneva, we showed that a natural consortium of bacteria leads to the inactivation of both CVA9 and E11 serotypes. After isolation of 136 bacterial isolates from the lake water, we observed that the two serotypes differed with respect to their susceptibility to inactivation by the different bacterial isolates. Measurement of the activities of different protease classes produced by each bacterial isolate furthermore revealed that MMP activity, and to a lesser extent serine protease activity, were associated with virus inactivation. Consequently, when lake water was treated with an MMPs inhibitor, the inactivation of both serotypes was reduced or completely inhibited. Overall, this study highlights the important role of bacterial extracellular proteases in controlling the persistence of enteroviruses in aqueous environments.

## Materials and methods

### Virus propagation and enumeration

Echovirus-11 (E11, Gregory strain, ATCC VR737) and Coxsackievirus-A9 (CVA9, environmental strain from sewage, kindly provided by the Finnish National Institute for Health and Welfare) stocks were produced by infecting sub-confluent monolayers of BGMK cells as described previously [7]. Viruses were released from infected cells by freezing and thawing the culture flasks three times. To eliminate cell debris, the suspensions were centrifuged at 3000 × *g* for 5 min. Each stock solution was stored at −20 °C until use. Infectious virus concentrations were enumerated by a most probable number (MPN) infectivity assay as described in the Supplementary Information. The assay limit of detection (LoD), defined as the concentration corresponding to one positive cytopathic effect in the lowest dilution of the MPN assay under the experimental conditions used, corresponding to 2 MPN/mL.

### Inactivation of enteroviruses by bacterial consortia from lake water

To study the inactivation of CVA9 and E11 by a bacterial consortium from lake water, four surface water samples were collected from Lake Geneva (Ecublens, Switzerland) during the summer 2021. Each sampling event was conducted on warm and sunny days, to minimize biological variation. Immediately after sampling, large particles of the sample were removed by filtering 500 mL of water on a 8 μm nitrocellulose filter membrane (Merck Millipore, Cork, Ireland). The sample was then filtered through a 0.8 μm nitrocellulose filter membrane (Merck Millipore) to remove large microorganisms such as protists. The resulting water sample corresponds to the bacterial fraction used to study virus inactivation.

For inactivation experiments, each virus was spiked into individual 1 mL aliquots of fractionated lake water to a final concentration of 10^6^ MPN/mL, and samples were incubated for 48 h at 30 °C without shaking. Duplicate experiments were conducted for each virus and each lake water sample. Experiments to control for thermal inactivation were conducted using the same procedure but by replacing the fractionated lake water with sterile milliQ water. Viral infectivity at times 0 h and 48 h was determined by MPN as described above. Virus decay was calculated as log_10_ (C/C_0_), where C is the residual titer after 48 h of incubation, and C_0_ is the initial titer. The experimental LoD was approximately 5-log_10_.

These same experiments were conducted for three new water samples in the presence of four protease inhibitors with the following final concentrations: E64—10 μM (E3132, Sigma–Aldrich, Saint-Louis, MO, USA), GM6001—4 μM (CC1010, Sigma–Aldrich), Chymostatin—100 μM (C7268, Sigma–Aldrich), and PMSF—100 μM (P7626, Sigma–Aldrich). Each inhibitor was added to 1 mL of fractionated lake water, vortexed for 30 seconds, and incubated at room temperature for 15 min, before adding the two viral strains under the same conditions as described above.

### Bacterial isolation, cultivation, and storage

Bacteria were isolated from two water samples from Lake Geneva’s Ecublens beach, taken in November 2019 (Fall, 89 isolates) and May 2020 (Spring, 47 isolates). Bacteria recovery was performed on R2A agar plate (BD Difco, Franklin Lakes, NJ, USA) as described previously [15]. Briefly, successive dilutions from 10^−1^ to 10^−5^ were carried out in sterile water for each sample. For each dilution, a volume of 1 mL was deposited on three separate R2A plates, before being incubated at 22, 30, and 37 °C. After 5 days of incubation, each colony was picked and enriched on a new R2A plate. To ensure purity, each isolate was successively plated five times on R2A plate and incubated at the same temperature as the initial isolation. Each purified isolate was cryopreserved in R2A / 20% glycerol at −80 °C. The isolates were named based on the water body (Lake (L)), isolation temperature, and the isolation order (L-T°C-number).

### Bacterial identification

The identification of each isolate was performed by 16 S rRNA gene sequencing using the pair of primers 27 F (5’- AGA GTT TGA TCM TGG CTC AG- 3’, Microsynth AG, Balgach, Switzerland) / 786 R (5’- CTA CCA GGG TAT CTA ATC – 3’, Microsynth AG), following a methodology previously described [15]. The thermocycling conditions and the purification of PCR products are described in the Supplementary Information. The complete list of isolated bacteria and associated accession numbers is given in Supplementary Table 1.

### Phylogenetic inference and metadata visualization

The consensus from 16 S rRNA gene sequences of the 136 isolates was aligned using the MUSCLE algorithm [16]. The phylogenetic analysis of 566 bp aligned sequences from the V2-V4 16 S rRNA gene regions (Positions: 152–717) was performed using Molecular Evolutionary Genetics Analysis X software [17]. Phylogeny was inferred by maximum likelihood, with 1000 bootstrap iterations to test the robustness of the nodes. The resulting tree was uploaded and formatted using iTOL [18].

### Virus incubation with bacterial isolates

For the preparation of the bacteria before co-incubation, each one was first cultured on R2A agar for 48 h at their initial isolation temperature. Overnight suspensions of each bacterial isolate were grown in R2A broth at room temperature under constant agitation (180 rpm). For co-incubation experiments, 200 μL of each bacterial suspension were mixed with 100 μL of a 10^5^ MPN/mL stock of E11 or CVA9. Then, each condition was supplemented with 600 μL of R2A broth. Incubation was carried out for 96 h at room temperature, without shaking. At the end of the co-incubation, each tube was centrifuged for 15 min at 9000 × *g* (4 °C) to eliminate bacteria, and the residual infectious viral titer was enumerated by MPN assay as described above [7]. Each co-incubation experiment was carried out in triplicate. Control experiments were performed under the same conditions but using sterile R2A. Virus decay was quantified as log_10_ (C_exp_/C_ctrl_), where C_exp_ is the residual titer after a co-incubation for 96 h, and C_ctrl_ is the titer after incubation of the virus in sterile R2A for 96 h. The experimental LoD was 3-log_10._

### Protease activity measurement using casein and gelatin agar plates

Casein agar was prepared as follows: 20 g of skim milk (BD Difco), supplemented with 1 g glucose were reconstituted with 200 mL of distilled water. Likewise, a 10% bacteriological agar solution was prepared in a final volume of 200 mL. Finally, a solution consisting of 0.8% NaCl, 0.02% KCl, 0.144% Na_2_HPO_4_, and 0.024% KH_2_PO_4_ was reconstituted in 600 mL of water. All solutions were autoclaved for 15 min at 110 °C. The solutions were mixed, and 25 mL were poured into each Petri dish. Gelatin agar was composed of 0.4% peptone, 0.1% yeast extract, 1.5% gelatin and 1.5% bacteriological agar. The mixture was autoclaved 15 min at 120 °C, and 25 mL of medium was poured into each Petri dish.

For each isolate, an overnight suspension was performed in R2A broth at room temperature, before spotting 15 μL of each suspension at the center of both gelatin and casein agar plates. Each plate was incubated at 22, 30, or 37 °C for 72 h, depending on the initial isolation temperature of the bacteria. Casein-degrading activity (*cas*), which is exerted by many different protease classes, and gelatin-degrading activity (*gel*), which is mostly caused by MMPs, were revealed by a hydrolysis halo around the producing bacteria. Hydrolysis diameters were measured in millimeters (mm) to report the extent of the proteolytic effect of each strain on both substrates.

### Protease activity quantification in cell-free supernatant

Using the same bacterial suspensions as for bacterial/virus co-incubation, 200 μL of each suspension was inoculated into 600 μL of R2A broth and incubated without shaking for 96 h at room temperature. Each culture was centrifuged for 15 min at 9000 × *g* at 4 °C. The resulting cell-free supernatants (CFS) were stored at −20 °C until use. For each CFS, protease activity was measured using the Protease Activity Assay Kit (ab112152, Abcam, Cambridge, UK), which measures general protease activity (*pgen*) except MMPs, and the MMP Activity Assay Kit (ab112146, Abcam), which selectively measures MMP activity (*mmp*). Briefly, for the Protease Activity Assay kit, 50 μL of the substrate was added into each well of a dark-bottom plate containing 50 μL of each CFS. Standard trypsin provided by the kit was used as a positive control. For the MMP Activity Assay kit, 50 μL of each CFS was incubated with 50 μL of 2 mM APMA for 3 h at 37 °C, prior to the activity test. Collagenase I (C0130, Sigma–Aldrich) was used as a positive control. R2A broth was used as a negative control for each assay. Protease activity was measured at time 0 and after 60 min, using a Synergy MX fluorescence reader (BioTek). The excitation and emission wavelengths were set to 485 and 530 nm, respectively. The emitted fluorescence, generated by proteolytic cleavage of the substrate of each kit, was calculated as follows: ∆_RFU_ = RFU (60 min) − RFU (0 min). Proteolytic activity was calculated in mmol/min/μL based on the emitted fluorescence measured for trypsin and collagenase I at known proteolytic activities.

### Data analysis

Statistical analyses to compare inactivation data were performed by one-way *t*-test or one-way ANOVA with Dunnett’s post-hoc test in GraphPad Prism v.9. An alpha value of 0.05 was used as a threshold for statistical significance. For each dataset we confirmed that data were normally distributed.

To analyze a potential correlation between protease activity and viral decay, the decay values for each virus strain was related to the four protease activity tests of this study using a scatterplot combined with a Kernel density estimation. The analyses were performed with R v.3.6.1 using the SmoothScatter function of the R Base package.

A Left-Censored Tobit model (CTM) with mixed effects was chosen to investigate interactions between protease activity and the decay measured for each virus strain. Briefly, the CTM with mixed effect was chosen for three reasons: (1) The protocol used to measure viral decay had a limit of quantification of −3-log_10_, and 152 measurement points reached the detection limit, requiring the use of this value as the left-censored value of the model; (2) The two virus strains used in the study showed distinct responses after exposure to environmental bacteria, preventing the use of a multiple linear regression model; (3) Among biological replicates of co-incubation experiments, inactivation variability was observed, suggesting the concomitant action of random biological effects (e.g., production of other compounds than proteases by bacteria, or differences in protease production rate between replicates for each bacterial isolate). The resulting statistical model was then formulated as follows:

$$\log \left( {\frac{{C_{{{{{{\mathrm{exp}}}}}}}}}{{C_{{{{{{\mathrm{ctrl}}}}}}}}}} \right) =	 \; \beta _0 + \beta _1\;{\rm I}_{{{{{{{{\mathrm{virus}}}}}}}}_i = 2} + \beta _2\sqrt {\left[ {pgen} \right]_i} + \beta _3\sqrt {\left[ {mmp} \right]_i} + \beta _4\sqrt {\left[ {cas} \right]_i} \\ 	+ \beta _5\sqrt {\left[ {gel} \right]_i} + \beta _6I_{{{{{{{{\mathrm{virus}}}}}}}}_i = 2}\sqrt {\left[ {pgen} \right]_i} + \beta _7I_{{{{{{{{\mathrm{virus}}}}}}}}_i = 2}\sqrt {\left[ {mmp} \right]_i} \\ 	+ \beta _8I_{{{{{{{{\mathrm{virus}}}}}}}}_i = 2}\sqrt {\left[ {cas} \right]_i} + \beta _9I_{{{{{{{{\mathrm{virus}}}}}}}}_i = 2}\sqrt {\left[ {gel} \right]_i} + \alpha _{{{{{{{{\mathrm{id}}}}}}}}_i} + \varepsilon _i$$

$${{{\mbox{where}}}}\; \log \left( {\frac{{C_{{{{{{\mathrm{exp}}}}}}}}}{{C_{{{{{{\mathrm{ctrl}}}}}}}}}} \right) = \left\{ {\begin{array}{*{20}{c}} { - 3} & {{{{{{{{\mathrm{if}}}}}}}}\;{{{{{{{\mathrm{log}}}}}}}}\left( {\frac{{C_{{{{{{\mathrm{exp}}}}}}}}}{{C_{{{{{{\mathrm{ctrl}}}}}}}}}} \right) \le - 3} \\ {{{{{{{{\mathrm{log}}}}}}}}\left( {\frac{{C_{{{{{{\mathrm{exp}}}}}}}}}{{C_{{{{{{\mathrm{ctrl}}}}}}}}}} \right)} & {{{{{{{{\mathrm{otherwise}}}}}}}}} \end{array}} \right.$$

$$\alpha _{{{{{{{{\mathrm{id}}}}}}}}_i}\sim {{{{{{{\mathrm{i}}}}}}}}.{{{{{{{\mathrm{i}}}}}}}}.\;{{{{{{{\mathrm{d}}}}}}}}.\;{\rm N}\left( {0,\;\sigma _{{{{{{{{\mathrm{id}}}}}}}}}^2} \right)$$

$${{{{{{{\mathrm{for}}}}}}}}\;i \in \left\{ {1,2, \ldots } \right\}$$

for which *β*_0_ defines the model intercept, $\beta _1{\rm I}_{{{{{{{{\mathrm{virus}}}}}}}}_i = 2}$ corresponds to the main effect of the virus factor on the viral decay, $\beta _2,\;\beta _3,\;\beta _4,\;{{{{{{{\mathrm{and}}}}}}}}\;\beta _5$ corresponds to the main effects of the different protease activity measurements on viral decay, $\beta _6I_{{{{{{{{\mathrm{virus}}}}}}}}_i = 2},\;\beta _7I_{{{{{{{{\mathrm{virus}}}}}}}}_i = 2},\;\beta _8I_{{{{{{{{\mathrm{virus}}}}}}}}_i = 2},{{{{{{{\mathrm{and}}}}}}}}\;\beta _9I_{{{{{{{{\mathrm{virus}}}}}}}}_i = 2}$ corresponds to the interaction effects between each of these variables and the viral decay, $\alpha _{{{{{{{{\mathrm{id}}}}}}}}_i}$ corresponds to the mixed effect of the model and $\varepsilon _i$ corresponds to the error term of the model. The selection of the model is further detailed in the Supplementary Information (Supplementary Material and Figs. S1 and S2**)**.

The full dataset included in the correlation analysis and the CTM is provided in Supplementary Table 2. A description of the variables used is given in the Supplementary Information. The dataset was analyzed using the censReg package in R [19]. The R code is given in the Supplementary Information.

## Results

### Bacterial consortia from lake water reduce the infectivity of E11 and CVA9 to different extents

**Fig. 1:**
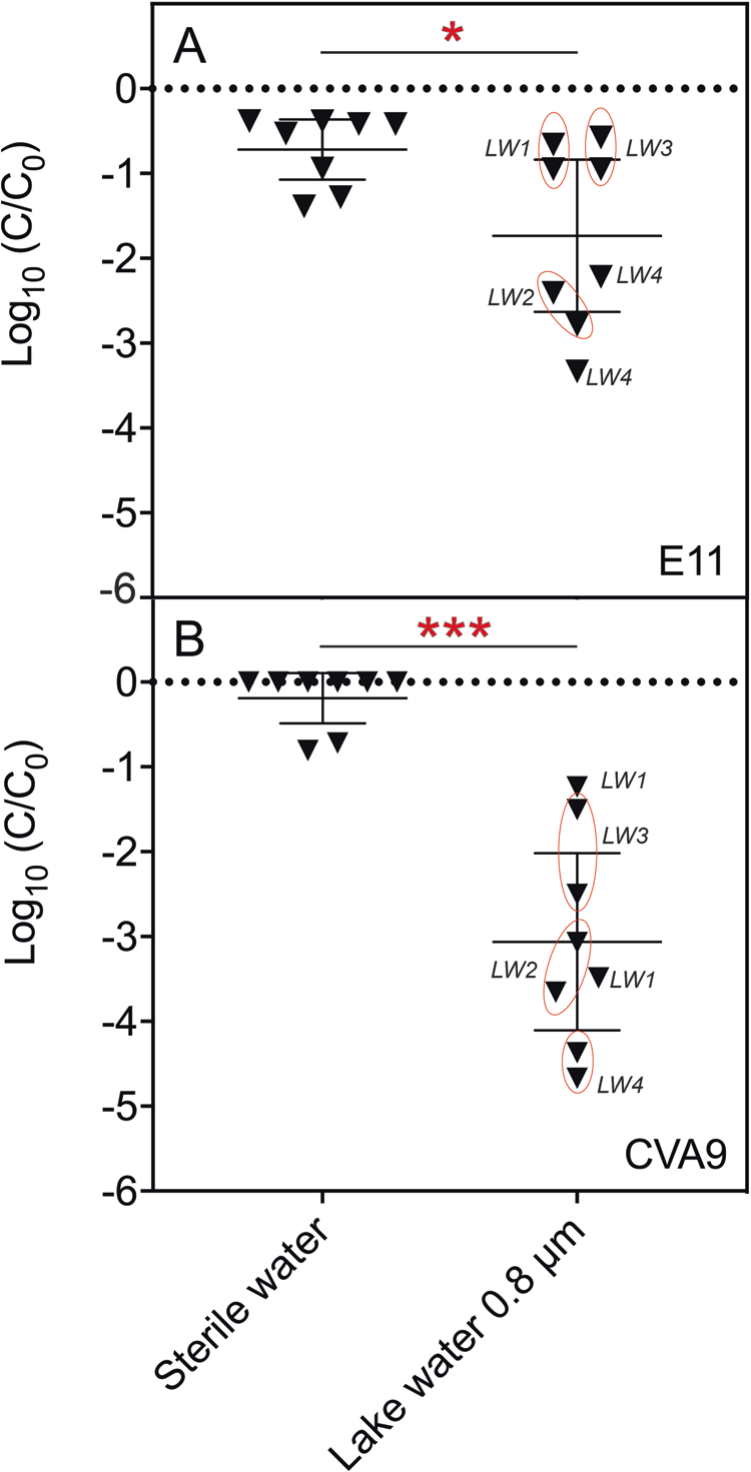
Bacterial consortia from lake water reduce the infectivity of E11 and CVA9 to different extents. **A** Inactivation of Echovirus-11 (E11). **B** Inactivation of Coxsackievirus-A9 (CVA9). An initial titer of 10^6^ MPN/mL was used for each virus strain. Each serotype was incubated independently in the same water fraction (<0.8 μm) for 48 h at 30 °C. Experiments were conducted with four biological replicates with two points of measurement per sample (LW1-LW4). Sterile water was used as negative control of inactivation. Viral decay in the control and in lake water was compared by *t*-test (* = *p*-value < 0.05; *** = *p*-value < 0.0005).

To investigate the impact of an environmental bacterial consortium on the extra-host inactivation of enteroviruses, we measured the effect of incubating two viral serotypes (E11, CVA9) in 0.8 µm-filtered lake water (Fig. 1). Infectivity measurement performed after 48 h of incubation showed that both serotypes were significantly inactivated compared to the control (*p*-values = 0.0002 (CVA9) and 0.0340 (E11). Furthermore, CVA9 was more strongly inactivated than E11, regardless of the water sample considered (mean inactivation CVA9: 3-log_10_; mean inactivation E11: 1.8-log_10_). The average inactivation of each serotype in sterile control samples corresponded to (E11 = 0.7-log_10_ (E11) and 0.2-log_10_ (CVA9). For E11, we observed that two lake water samples (LW1, LW3), did not cause inactivation beyond that observed in the control (Fig. 1A). On the other hand, all lake water samples induced at least 1.2-log_10_ decay of CVA9, this observation being up to 4.8-log_10_ depending on the sample (Fig. 1B). This experiment suggests that the inactivation of enteroviruses by lake water bacteria depends on the serotype studied as much as on the composition of bacteria in each water sample.

### All bacterial classes isolated from lake water cause inactivation of CVA9 and E11 but the antiviral activity differs between isolates of a given class

**Fig. 2:**
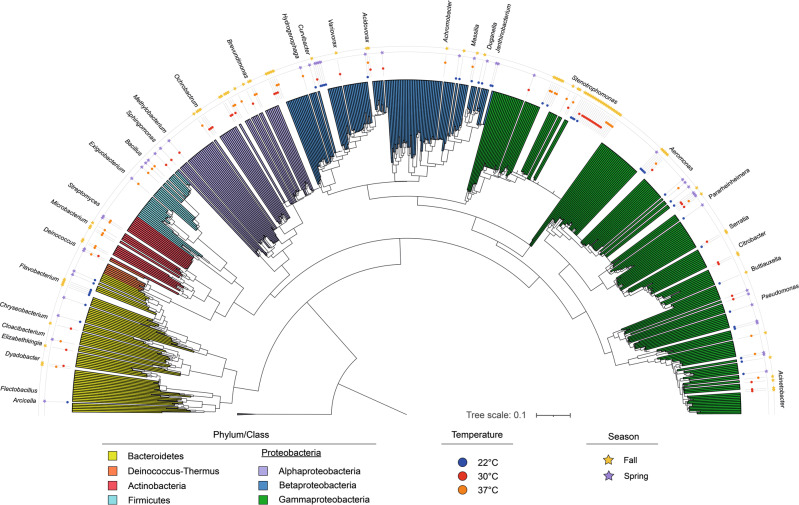
Phylogenetic distribution of the bacterial isolates used in this study. The phylogeny was inferred using the Maximum Likelihood method. The reliability of the inferred phylogeny was tested with the Bootstrap method, applying 1000 replicates to the test. Bootstrap values above 0.6 were displayed on the branches. The isolation and growth temperatures of the bacteria, as well as the sampling season, were specified as metadata. The reference strains selected to anchor the tree belong to the genus *Methanosarcina* (*M. vacuolata* Z-761, *M. barkeri* MS, *M. flavescent* E03.2, *M. spellaei* DSM 26047, *M. sicilian* T4/M). The phylogenetic reconstruction was performed using MEGA V10.1.8. The tree formatting and metadata insertion were done using iTOL.

To identify the role of different bacteria in the inactivation of E11 and CVA9, a collection of bacterial isolates from Lake Geneva was created. A total of 136 bacterial isolates were recovered and identified by 16 S rRNA gene sequencing. The resulting sequences were subjected to phylogenetic analysis to reflect the diversity of isolates in the collection (Fig. 2). All strains are distributed within the phyla *Bacteroidetes* (14/136), *Actinobacteria* (7/136), *Proteobacteria* (108/136), *Deinococcus-Thermus* (2/136), and *Firmicutes* (5/136). The phylum *Proteobacteria* is mostly represented by the class *Gammaproteobacteria* (67/108), and to a lesser extent by the classes *Betaproteaobacteria* (21/108) and *Alphaproteobacteria* (20/108). A total of 31 bacterial genera were identified, among which *Stenotrophomonas* sp. is the most abundant one, representing 24% of the isolates.

**Fig. 3:**
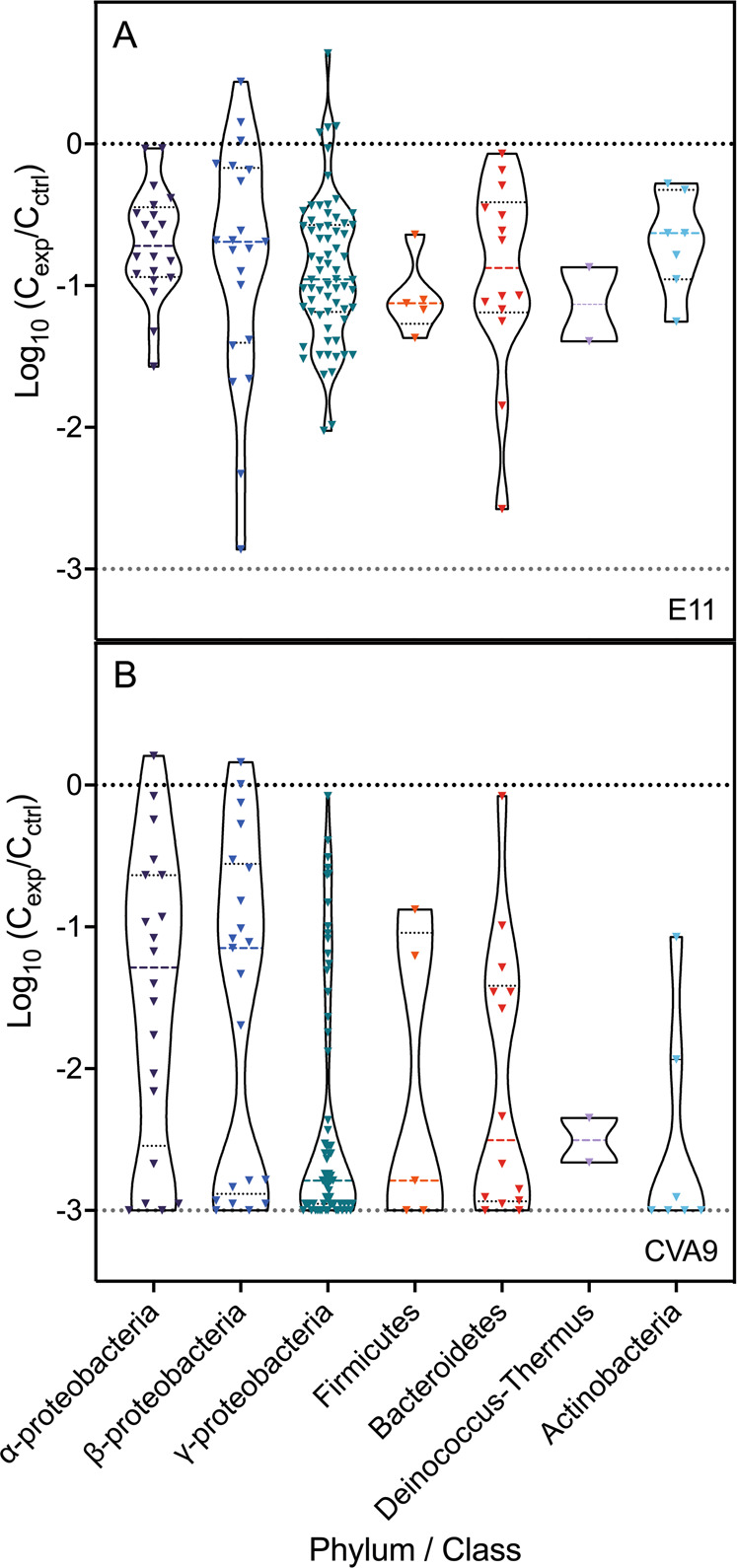
Infectivity of E11 and CVA9 are reduced by differing extents during co-incubation with a taxonomic diversity of lake bacterial isolates. Co-incubation experiments with bacterial isolates and E11 (**A**) or CVA9 (**B**) were conducted for 96 h at room temperature. Each point represents the average decay obtained from the triplicate experiments for a given combination of virus and bacterial isolate. In each violin, the thick dotted line represents the median of the decay values obtained in the phylum or class studied and the thin dotted lines indicate the interquartile range. The dotted line at −3-log_10_ represents the detection limit of the assay (LoD).

Each bacterial isolate was separately co-incubated for 96 h with CVA9 and E11 and the resulting infectivity loss was measured (Fig. 3). The inactivation of E11 by the isolated bacteria was mostly restricted to <1-log_10_, with the highest decays in infectious titers induced by co-incubation with bacteria belonging to *Proteobacteria* or *Bacteroidetes* phyla (Fig. 3A). In contrast, we found that CVA9 was readily inactivated by the majority of isolates, regardless of the taxonomic classification of the bacteria studied (Fig. 3B). A high proportion of bacterial isolates induced inactivation below the detection limit of the virus (2 MPN/mL). However, a subset of isolates exerted no effect on CVA9. Therefore, it seems that there are differences in the antiviral action targeting CVA9 within each phylum or class, probably at the level of the bacterial genus or species. Overall, these results demonstrate that CVA9 and E11 are not inactivated to the same extent by the same bacterial isolates.

### More than 90% of bacterial isolates inducing at least 1-log_10_ viral inactivation produce one or several types of proteases

**Fig. 4:**
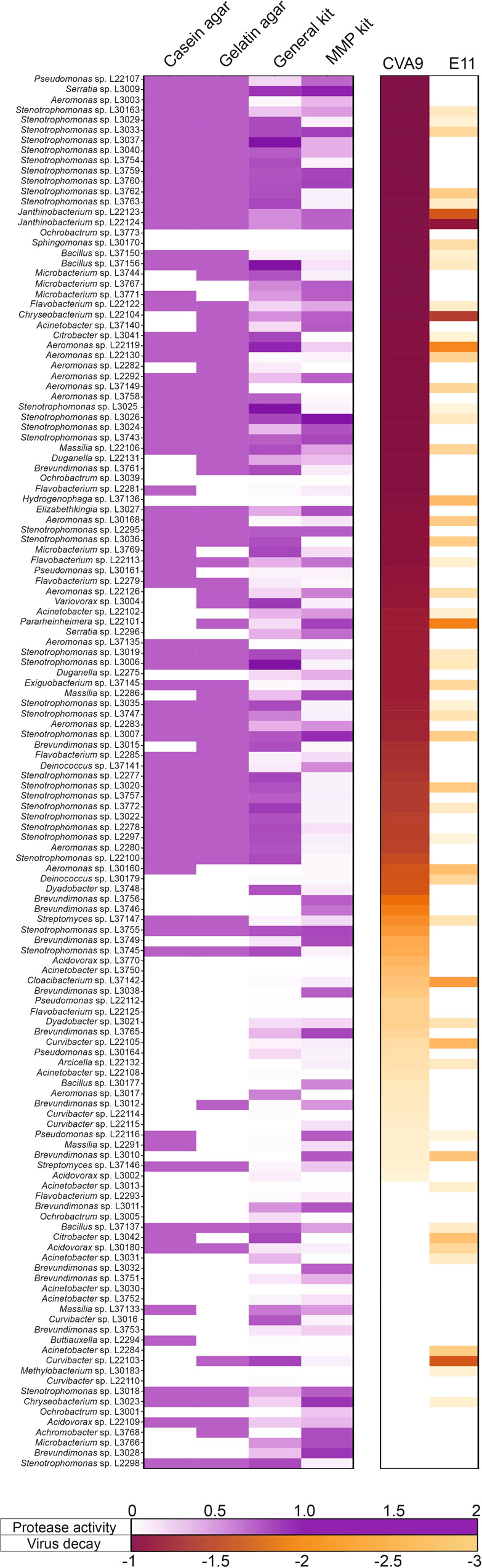
Most bacterial isolates (>90%) inducing more than 1-log inactivation for both virus strains produce at least one type of protease. Casein and gelatin degradation by each bacterial isolate was investigated by proteolysis halo measurement (mm) on milk and gelatin agar plates. Protease activity in CFS was measured by fluorescence using a general protease activity kit (for all proteases family except MMPs) and a metalloproteinase activity kit. CVA9; E11: viral inactivation values measured after a co-incubation with each bacterial isolate. Values are means of triplicate experiments. The heatmap from white to purple indicates the normalized protease activity for each test performed. The heatmap from yellow to red indicates the decay values measured for each viral strain.

To explore the production of extracellular proteases by the bacterial isolates, we tested the ability of each isolate to degrade either casein or gelatin. In addition, the general protease activity and the specific MMP activity were quantified in each CFS. Each protease profile was subsequently compared with the decay values obtained for each of the two virus strains E11 and CVA9 (Fig. 4). Of all the bacterial strains isolated, 110 induced decay of more than 1-log_10_ of CVA9 infectivity. Among those, 104 (94.5%) showed a positive signal for at least one of the four protease activity tests performed. Only 52 bacterial isolates induced decay of E11 of more than 1-log_10_, and 50 of them (96.2%) showed a positive signal for one of the protease tests performed. Of all the bacterial isolates tested, five belonging to the genera *Janthinobacterium*, *Chryseobacterium*, *Aeromonas,* and *Pararheinheimera* induced decay of at least 2-log_10_ for both serotypes. While the three isolates affiliated with the genera *Janthinobacterium* and *Aeromonas* exhibited protease activity in all assays used, the two isolates of *Chryseobacterium* and *Pararheinheimera* only showed a gelatin-degradation activity and activity related to the presence of MMPs. The isolates belonging to *Stenotrophomonas*, the genus most represented in the collection (33/136), showed a positive response on casein agar, on gelatin agar and using the general protease activity kit. However, only 14 of these isolates produced protease activity measurable by the specific MMP kit. Of these 33 bacterial isolates, 31 induced inactivation of CVA9, while only four isolates inactivated E11. These results demonstrate that a diversity of proteases is produced in the microenvironment of lake bacteria, supporting a possible role for proteases in the viral decay of E11 and CVA9. However, a subset of bacterial isolates induced inactivation without detectable protease activity. Conversely, several isolates produced proteases but did not induce inactivation. Protease production alone is thus not a sufficient condition for virus inactivation.

### Metalloprotease activity is most predictive of the inactivation of E11 and CVA9

**Fig. 5:**
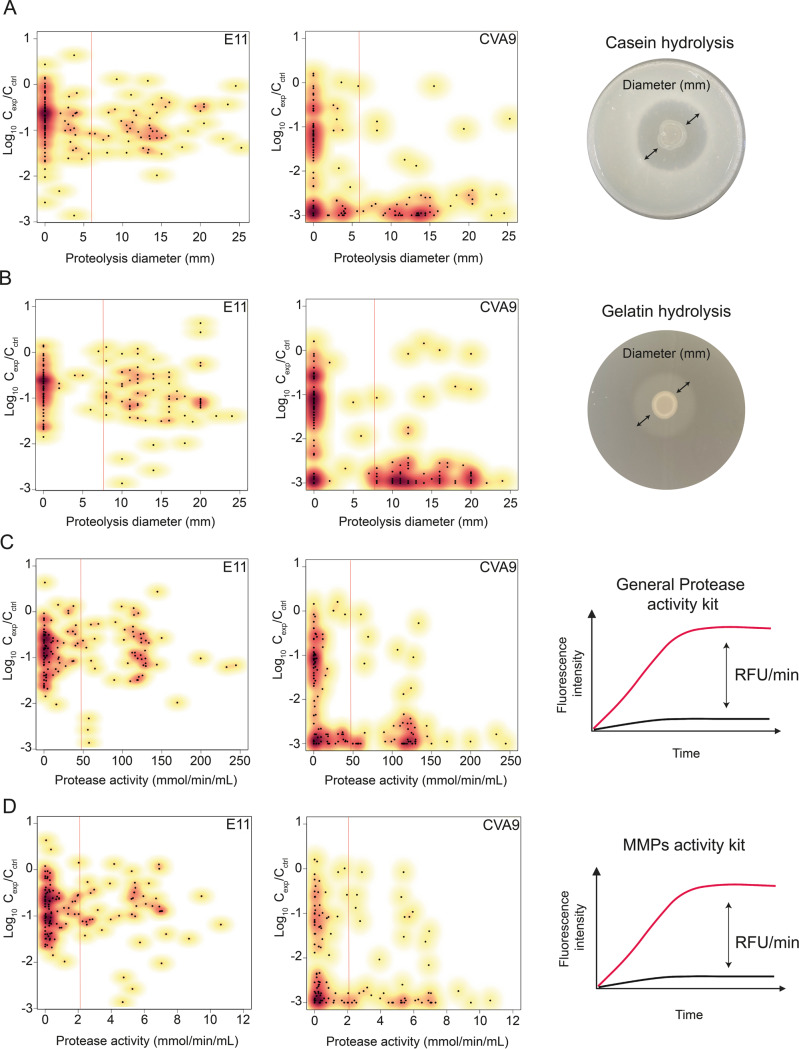
Absence of a linear correlation between virus decay and the presence of each type of protease. **A, B** Distribution of viral decay according to the proteolysis diameter (mm) measured for each bacterial isolate **A** on casein agar and **B** on gelatin agar. **C, D** Distribution of viral decay according to the quantitative protease activity measured in CFS by fluorescence (nmol/min/mL) **C** using a general activity kit and **D** using an MMPs specific kit. Red vertical lines represent the average protease activity measured by each assay. The kernel density is represented from yellow to red, indicating low to high density of data points, respectively.

To investigate a possible correlation between protease production and inactivating activity on E11 and CVA9, protease activity values derived from the four assays used in this study were quantitatively compared to the decay of E11 and CVA9 (Fig. 5). No linear correlation was found between the presence of any protease in the extracellular environment of bacteria and the inactivation of a particular serotype (Fig. 5A–D). While most bacteria that strongly inactivate E11 (≥2-log_10_) showed no evidence of casein-degrading proteases (Fig. 5A), the same effect coincided strongly with MMP and gelatin-degrading proteases activities (Fig. 5B, D). Among the five bacterial candidates leading to such inactivation, all of them show an above-average activity (>7 mm) for gelatin hydrolysis, and four of them were also associated with above-average MMPs activity measured by fluorescence (2000 nmol/min/mL) (Fig. 5B, D). In contrast, a large part of the bacteria causing >2-log_10_ inactivation of CVA9 exhibited above-average (>6 mm) casein-degrading activity (Fig. 5A). Because many different proteases can induce casein degradation, the inactivation of CVA9 also coincided with high measures of general protease activity by fluorescence (>50,000 nmol/min/mL) (Fig. 5C). Above-average protease activity related to MMPs was also found in many isolates causing rapid degradation of CVA9. However, a small cluster of isolates that inactivated CVA9 with the same magnitude did not produce measurable MMP activity (Fig. 5B, D, bottom left corner).

To support these observations, all the data collected were analyzed using a Left-Censored Tobit Model (CTM) with mixed effects. Among the 816 decay observations, 152 values were censored at −3-log_10_, corresponding to the detection limit of the assay used in the screening. The analysis of the main effect of the enzymatic activity measured by the four assays used indicated that an increase in protease activity coincided with an increase in viral decay, but the significance of this observation was greatest for gelatin-degrading proteases (*gel*; decay unit: −0.126; *p-*value: 9.31E-09) and matrix metalloproteases (*mmp*; decay unit: −0.011; *p*-value: <2E-16) (Table 1). Correspondingly, the likelihood-ratio test showed that the prediction only depended on protease activity as quantified by the general kit (*pgen: p*-value = 0.002; χ2 = 10.043) and the MMP kit (*mmp*: *p*-value = 4.24e-06; χ2 = 21.151). The model furthermore revealed a significant interaction between virus type and *pgen* (0.002; *p*-value = 1.36E-06) and virus type and *mmp* (0.011; *p*-value = 2E-16). Finally, the mixed effect of the model predicted variability of 0.982-log_10_ in the decay values ($\alpha _{{{{{{{{\mathrm{id}}}}}}}}_i}$), indicating that other environmental factors, such as thermal inactivation, are involved in the inactivation of CVA9 and E11 under our experimental conditions.

**Table 1:** Statistical output of the censored Tobit regression model with mixed effects, fitting virus decay as a function of protease activity measured by different assays and their interaction with virus type.

					Likelihood-ratio test	
Variables		Estimate	Std. error (CI 95%)	*p*-value	*X*2	*p*-value
Intercept		−0.868	0.043	<2.00E-16		
Virus factor		−0.241	0.064	1.00E-04		
Casein hydrolysis	sqrt(*cas*) main effect	−0.073	0.023	0.001		
	sqrt(*cas*) interaction	0.002	0.029	0.945	0.004	0.951
Gelatin hydrolysis	sqrt(*gel*) main effect	−0.126	0.022	9.31e-09		
	sqrt(*gel*) interaction	−0.005	0.029	0.861	0.030	0.863
General protease activity kit	sqrt(*pgen*) main effect	−0.001	2.00E-04	0.002		
	sqrt(*pgen*) interaction	0.002	3.00E-04	1.36E-06	10.043	0.002
MMP activity kit	sqrt(*mmp*) main effect	−0.011	0.001	<2.00E-16		
	sqrt(*mmp*) interaction	0.011	0.001	<2.00E-16	21.151	4.24e-06
Error term		−0.190	0.019	<2.00E-16		
αidi		−0.983	0.019	<2.00E-16		

Together, these results indicate that the viral decays measured for E11 and CVA9 depend preferentially on the activity of MMPs, but that proteases belonging to other classes (e.g., serine, cysteine, or aspartic proteases) also contribute to the inactivation.

### MMPs and serine proteases produced by a consortium of lake bacteria are involved in E11 and CVA9 inactivation

**Fig. 6:**
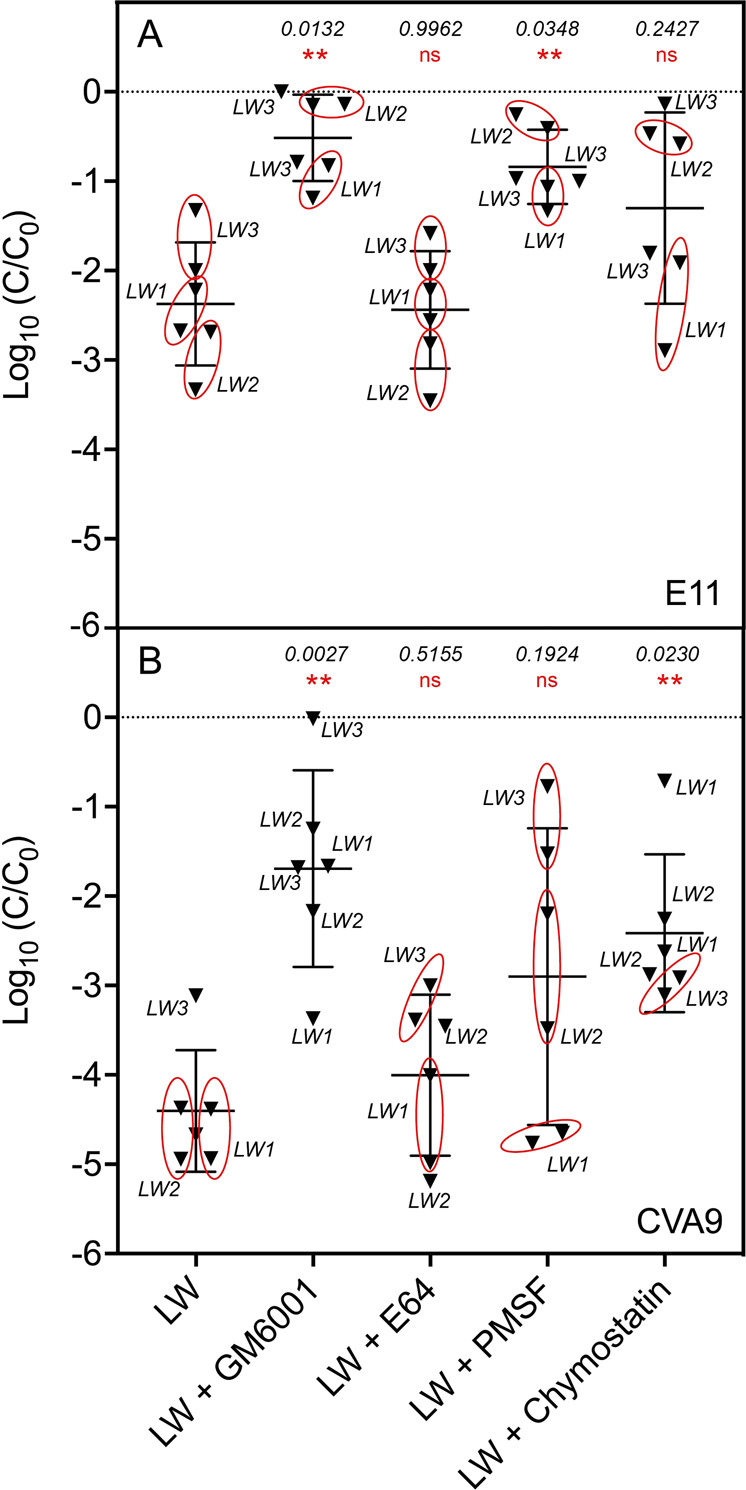
Addition of MMP and serine proteases inhibitors to a bacterial consortium from lake water reduces antiviral activity. E11 (**A**) or CVA9 (**B**) were  incubated independently in the same water fraction (<0.8 μm) for 48 h at 30 °C, with and without specific protease inhibitors. GM6001 was used to inhibit MMPs, E64 was used to inhibit cysteine proteases, PMSF and Chymostatin were used to inhibit serine proteases. Experiments were conducted with three biological replicates (LW1-LW3) with two measurement points per sample. An initial titer of 10^6^ MPN/mL was used for both serotypes. Differences in viral decay were analyzed by one-way ANOVA analysis with post-hoc Dunnett test (ns: *p* > 0.1, *: 0.01 < *p* < 0.1, **: 0.01 < *p* < 0.001). Numbers in italic indicate resulting *p*-values.

To validate the significance of proteolytic events occurring naturally in lake water, the incubation of each virus with filtered lake water was repeated, though in the presence of four different protease inhibitors (Fig. 6). The addition of GM6001, a specific inhibitor of MMPs, completely suppressed the inactivation of E11 (*p*-value = 0.0132), and strongly reduced the inactivation CVA9 (*p*-value = 0.0027). In contrast, E64, which specifically inhibits cysteine proteases, showed no effect on either serotype (*p*-values = 0.9962 (E11) and 0.5155 (CVA9)). The serine protease inhibitor PMSF, an inhibitor targeting a diversity of serine proteases including trypsin and chymotrypsin, exerted a differential effect on the two serotypes. Specifically, the addition of PMSF induced a systematic reduction in E11 inactivation of 2-log_10_ (*p*-value = 0.0348), whereas for this effect was significant on average (*p*-value = 0.1924), yet highly variable for CVA9, ranging from 0 (LW1) to 4-log_10_ (LW3). Similarly, the use of chymostatin, a chymotrypsin-like protease inhibitor, induced a 2-log_10_ reduction in CVA9 inactivation (*p*-value = 0.0230), whereas this effect ranged from 0 (LW1) to 2.5-log_10_ (LW2) for E11 (*p*-value = 0.2427). These findings are consistent with the measured effect of inhibitors on the general protease activity of the lake water. Specifically, the inhibitors reducing virus decay (GM6001, PMSF, chymostatin) were also found to reduce the general protease activity of the samples, whereas E64 did not suppress virus decay nor yield a measurable decrease in protease activity (Supplementary Fig. S3). Overall, data showed that bacterial MMPs and serine proteases are involved in the inactivation of both E11 and CVA9 serotypes.

## Discussion

Enteroviruses are discharged into natural environments, but their fate in these environments requires further understanding. We previously showed that in lake water, enterovirus fate is governed by solar inactivation in the top layer of the water column, while microorganism-driven decay dominates below ~1 m, in particular during the warm season [7]. Yet the role of different microorganisms in regulating processes relevant to water quality remains understudied. In this work, we explored the ability of bacteria from Lake Geneva to inactivate the two enterovirus serotypes E11 and CVA9, emphasizing the importance of extracellular bacterial proteases in this process.

By incubating these two viruses in lake water containing indigenous bacteria, E11 and CVA9 showed an average loss of infectivity of 1.5-log_10_ and 3-log_10_, respectively, over the course of 2 days. The results also revealed that infectivity losses differed between water samples. While all samples were taken during dry and sunny weather, intermittent days with heavy rainfall between sampling events were noted. Weather variations are known to modify the level of nutrients available for microorganisms, naturally reshaping bacterial populations over short periods of time [20, 21]. Rainfall may thus contribute to the observed differences in virus decay between samples.

After creating a collection of bacteria from the same sampling site, the inactivation of E11 and CVA9 by each bacterial isolate was evaluated. The results demonstrate that CVA9 was strongly inactivated in the presence of several bacterial isolates affiliated with the five phyla identified in this study, namely *Proteobacteria*, *Bacteroidetes*, *Firmicutes*, *Deinococcus-Thermus,* and *Actinobacteria*. In contrast, E11 was only strongly inactivated by bacteria of the *Betaproteobacteria* and *Gammaproteobacteria* classes and the *Bacteroidetes* phylum. Therefore, the specific combination of the bacterial species and the virus serotype appears to be a key factor in understanding inactivation rates and mechanisms.

Our findings are consistent with prior reports on CVA9 inactivation by different bacterial species. A previous study has shown that *Bacillus subtilis* and *Pseudomonas aeruginosa* induced the inactivation of CVA9, whereas others, such as *Escherichia coli*, *Proteus vulgaris,* or *Salmonella* spp. showed no effect [10]. From the collection reported in this work, four bacterial isolates were affiliated with the genus *Bacillus* sp. and five with the genus *Pseudomonas* sp. Except for one *Bacillus* sp. isolate, all isolates of these two bacterial genera showed inactivation of serotype CVA9. Of these isolates, four also induced a 1-log_10_ decay in E11 infectivity under the same conditions.

The analysis of the proteolytic activities of each bacterial isolate in the collection revealed that most cultivable bacteria isolated herein produced proteases of several families. While some studies have shown the direct inactivation of enteroviruses by commercial proteases, we sought to identify the effect of the proteases produced by lake water bacteria on the persistence of E11 and CVA9. Inactivation was not directly proportional to any of the measures of protease activity used herein. Nevertheless, an analysis of the impact of different protease types on the decay of both viruses was performed using Tobit regression with mixed effect and showed that the activities of MMP and general protease were predictive of the inactivation of E11 and CVA9, with metalloproteases showing the greatest significance. While the proteases produced in this work were not characterized, a previous study assessed several serine proteases and MMPs with respect to their inactivating effect on CVA9 [10]. After 1 h of incubation at 37 °C, the authors found a net inactivation of CVA9 of 1.40-log_10_ and 1.78-log_10_ after treatment with the serine proteases subtilisin and trypsin, while the two other serine proteases used in their study (peptidase, lysozyme) did not show such an effect. Similarly, the addition of neutral protease (MMPs) induced a net inactivation of 2-log_10_ for this virus. Finally, the incubation of pronase with this virus, mimicking the co-presence of several types of proteases, induced the highest viral decay for CVA9 (2.3-log_10_).

To confirm the importance of these proteases on the observed inactivation, the use of specific inhibitors on a bacterial consortium from the lake showed that MMPs were indeed involved in the inactivation of E11 and CVA9. The treatment of water samples with two serine protease inhibitors also reduced inactivation, even if the effect was less pronounced. In contrast, we found no evidence that cysteine proteases contribute to inactivation. While MMPs and serine proteases are thus involved in the inactivation of enteroviruses, it is unclear whether serine proteases directly attack the capsid, or mediate MMP activity. Indeed, MMPs are known to adopt two main forms in solution, an inactive folded form (pro-MMP state) and an active unfolded form (MMP state), the transition between the two passages being activated by some serine or cysteine proteases [22, 23]. A synergistic action between different catalytic protease types is also consistent with earlier findings that protease mixtures (pronase) cause greater inactivation than individual protease classes alone [10].

For each experiment conducted on both viruses of this study, we consistently observed higher decay values for CVA9. Besides the fact that CVA9 may be the target of a broader range of proteases, this serotype is also described to have a 17-mers RGD peptide exposed at the C-terminus of the VP1 that may constitute a substrate for proteases. Whereas the optimal infectivity efficiency of BGMK cells by CVA9 depends on the recognition between the RGD motif and specific integrins [24], the cleavage of the external peptide by trypsin does not fully prevent the virus from a bypass of entry into these cells [25].

## Conclusion

Bacterial extracellular proteases naturally produced by Lake Geneva bacteria contribute to the control of enteroviruses. Inactivation rates differed between serotypes, implying that serotypes differ in their environmental persistence. While the results of this work emphasize the importance of MMPs in virus control, the action of serine proteases was also identified as relevant. Serine proteases may directly act on viral proteins, or they may act indirectly via the activation of bacterial pro-MMPs into active MMPs. To better understand the proteolytic inactivation mechanisms of enteroviruses and the resulting inactivation rates, work remains to be done on the protease/capsid recognition specificity, as well as on the characterization of the proteases that can lead to such inactivation. Nevertheless, this work provides the first overview of the proteolytic events occurring in the microenvironment of enteroviruses, identifying specific protease classes as potential proxies to monitor virus inactivation in surface waters.

## Supplementary information

Supplementary Material and Figures

Supplementary table 1

Supplementary table 2

## Data Availability

16 S rRNA gene sequences were deposited in the GenBank database under accession numbers OK501985 to OK502119. All raw data including protease activities and virus decay values are available in the Supplementary Information and at 10.5281/zenodo.6356976.
