# Supplementary material for: Bacterial matrix metalloproteases and serine proteases contribute to the extra-host inactivation of enteroviruses in lake water
Source: ISME J. 2022 May 11;16(8):1970–9. doi: 10.1038/s41396-022-01246-3 (PMC9296489; doi:10.1038/s41396-022-01246-3)
Supplement: Supplementary file 1 — Supplementary Material and Figures [file 41396_2022_1246_MOESM1_ESM.pdf]

Supplementary Information

Bacterial matrix metalloproteases and serine proteases contribute to the extra-host inactivation of enteroviruses in lake water

Marie-Hélène Corre[a], Virginie Bachmann[a #] and Tamar Kohn[a*]

[a] Laboratory of Environmental Chemistry, Environmental Engineering Institute (IIE), School of Architecture, Civil and Environmental Engineering (ENAC), Ecole Polytechnique Fédérale de Lausanne (EPFL), Lausanne, Switzerland

[*]Corresponding author: Tamar.Kohn@epfl.ch

[#] Current address: Metabolic Modelling Group, Nestlé Institute of Health Sciences, EPFL Innovation Park Building G, 1015 Lausanne

Supplementary material

Virus enumeration

Virus sample aliquots (100 µL) were diluted by 10^{-1} to 10^{-8} in MEM 2% FBS. Each dilution (100 µL, 5 replicates) was plated on a BGMK sub-confluent monolayer in a 96-well plate and was incubated at 37°C 5% CO_2. After 4 days, the number of wells with cytopathic effect for each dilution was recorded, and the resulting number of infectious viruses per sample was calculated using R [1].

Bacterial identification

For the amplification of the 16S gene by PCR, the DreamTaq Green PCR Master Mix kit (Thermo Scientific, Rockford, IL, USA) was used. The thermocycling program consisted of an initial denaturation of 2 min at 94°C, followed by 30 cycles comprising successively 30 seconds denaturation (94°C), 30 seconds annealing (50°C) and 1 min extension (72°C), followed by a final extension of the product for 5 min at 72°C. The purification of PCR products was done with the Invisorb® Fragment CleanUp kit (Invitek Molecular, Berlin, Germany), following the supplier's recommendations.

DNA sequencing was performed with the ABI Prism BigDye terminator v3.1 kit (Applied Biosystems™), following the kit recommendations. Precipitation of sequencing products was performed by adding 75 µL of 70% ethanol-0.5 mM $MgCl_2$ to each reaction. After 20 minutes of centrifugation at 3000x*g*, the products were washed by centrifugation (20 minutes, 3000x*g*) with 200 µL of 70% ethanol. The resulting pellets were resuspended in 25 µL formamide. Sequence analyses were done using a 3730xl DNA Analyzer (Applied Biosystems™). Each electropherogram was manually checked and cleaned using 4Peaks v1.8. Contigs were constructed with SerialCloner software (v2.6) before being compared to the 16S ribosomal RNA sequences database using BLASTn [2].

Description of data set presented in Supplementary Table 2

The dataset analyzed in the correlation analysis and the Left-Censored Tobit model contains 136 rows, represented by each bacterial isolate in this study. The dataset is composed of 23 columns, describing respectively: the code assigned to each bacterial isolate, the initial assignment number, the corresponding sample date, the initial isolation temperature, the proteolysis diameter on milk agar in millimeter, the proteolysis diameter on gelatin agar in millimeter, the bacterial genus identified, the closest species identified using the 16S reference database (NCBI), the decays measured for CVA9 (raw values, means, SD, 95% confidence interval), the decay measured in E11(raw values, means, SD, 95% confidence interval), the global protease activity measured in CFS, the specific metalloprotease activity measured in CFS (**Supplementary Table 2**).

R code for the Left-Censured Tobit Model with mixed effects

The model was implemented in R using the censReg library.

```
dd <- read.csv2("dataset.csv", header = TRUE,
                skip = 2, stringsAsFactors = F)

dd <- dd[, c(5,6, 10, 11, 12, 16, 17, 18, 22, 23)]
str(dd)
colnames(dd) <- c("cas","gel", "n1", "n2", "n3",
                "m1", "m2", "m3", "mmp", "pgen")
decay1 <- vector()
decay2 <- vector()
for ( i in 1:dim(dd)[1]){
  decay1 <- c(decay1, as.numeric(dd[i, c("n1", "n2", "n3")]))
  decay2 <- c(decay2, as.numeric(dd[i, c("m1", "m2", "m3")]))
}

cas <- as.numeric(rep(dd[ , "cas"], each = 3))
gel <- as.numeric(rep(dd[ , "gel"], each = 3))
mmp <- as.numeric(rep(dd[ , "mmp"], each = 3))
pgen <- as.numeric(rep(dd[ , "pgen"], each = 3))
virus <- factor(c(rep(1, dim(dd)[1]),
                  rep(2, dim(dd)[1])))
id <- factor(rep(1:(2*dim(dd)[1]), each = 3))
id
dat.org <- data.frame(id, c(decay1, decay2),
                  rep(cas, times = 2),rep(gel, times = 2),
                  rep(mmp, times = 2),
                  rep(pgen, times = 2), virus)

colnames(dat.org) <- c("id", "decay", "cas","gel",
                   "mmp", "pgen", "virus")
str(dat.org)
head(dat.org)
dat.org

library(censReg)
fit.censreg1 <- censReg( decay ~ virus * (sqrt(pgen) + sqrt(mmp) +
                     sqrt(cas) +sqrt(gel)), data = panel.dat,
                   method = "BHHH", left = -3)
drop1(fit.censreg1, test = "Chisq")
summary(fit.censreg1)
```

Selection of the statistical model

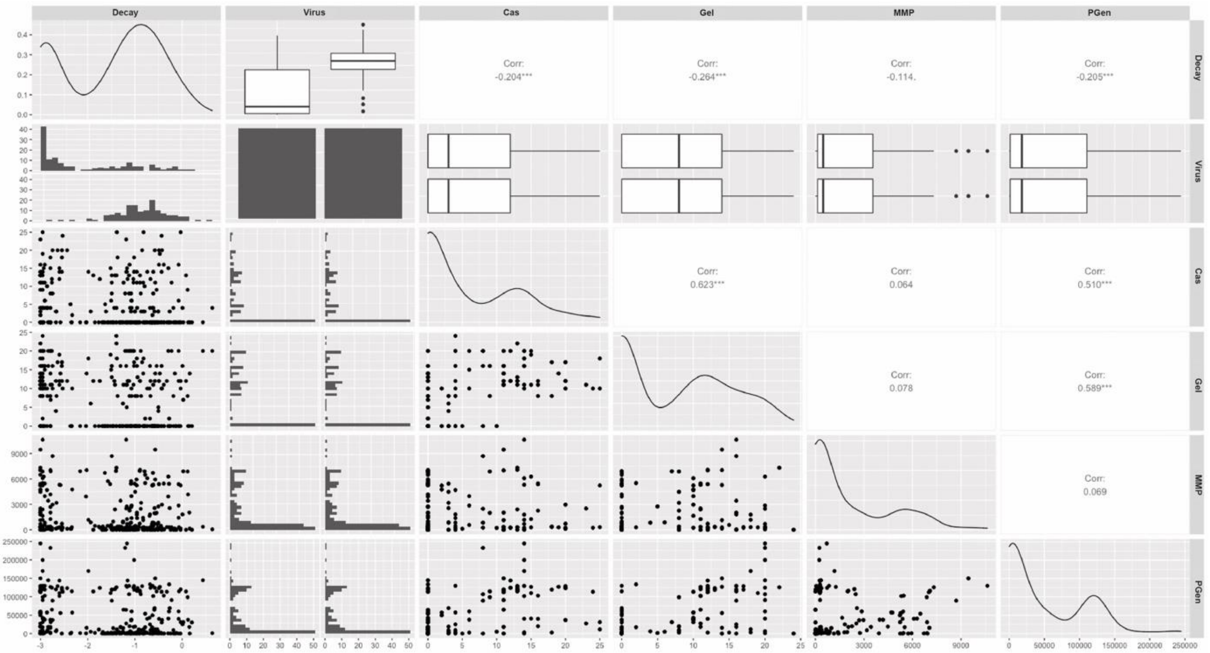

Figure S1: Overall analysis of the variables contained in the dataset of this study. *decay* corresponds to the numerical distribution of mean decay value measured for each virus (resulting from biological triplicate). *virus* is composed of the two viruses of the study (virus 1: CVA9, virus 2: E11). *cas* represents the numerical values of the diameters measured on milk agar. *gel* represents the numerical values of the diameters measured on gelatin agar. *mmp* represents the numerical values of the protease activity measured with the matrix metalloproteinase kit. *pgen* is the numerical value of the protease activity measured with the general kit. The overview graph was generated in R base using the library *GGally*.

As a first step, we analyzed the virus decay values as a function of the different experimental variables considered (*virus*, *cas*, *gel*, *mmp*, *pgen*). Correlation analysis indicated that the decay data did not follow a normal distribution (top left panel, **Figure S1**), but instead exhibited two types of decay responses. Decay distributions as a function of virus species (second panel in left column) also showed two distinct patterns, one of them with a large number of -3-$\log_{10}$ censored values (LoD). Given the large amount of left-censored data, a tobit regression was chosen for further statistical analysis.

The first tobit regression was performed using the function *fit.tobit* under the R library VGAM[3, 4], using the absolute value of the different explanatory variables as input. This

approach yielded a residual distribution that deviated from normality (**Figure S2.A**). In a next attempt, the same model was applied, but using the square root of each explanatory variable (*fit.tobit2*) rather than the absolute value. This approach slightly improved the log-likelihood (LL) of the model ($LL_{fit.tobit}$ =-306.315; $LL_{fit.tobit2}$ = -302.694). However, the residual analysis did not show significant differences compared to the analysis performed with the explanatory variables directly (**Figure S2.B**).

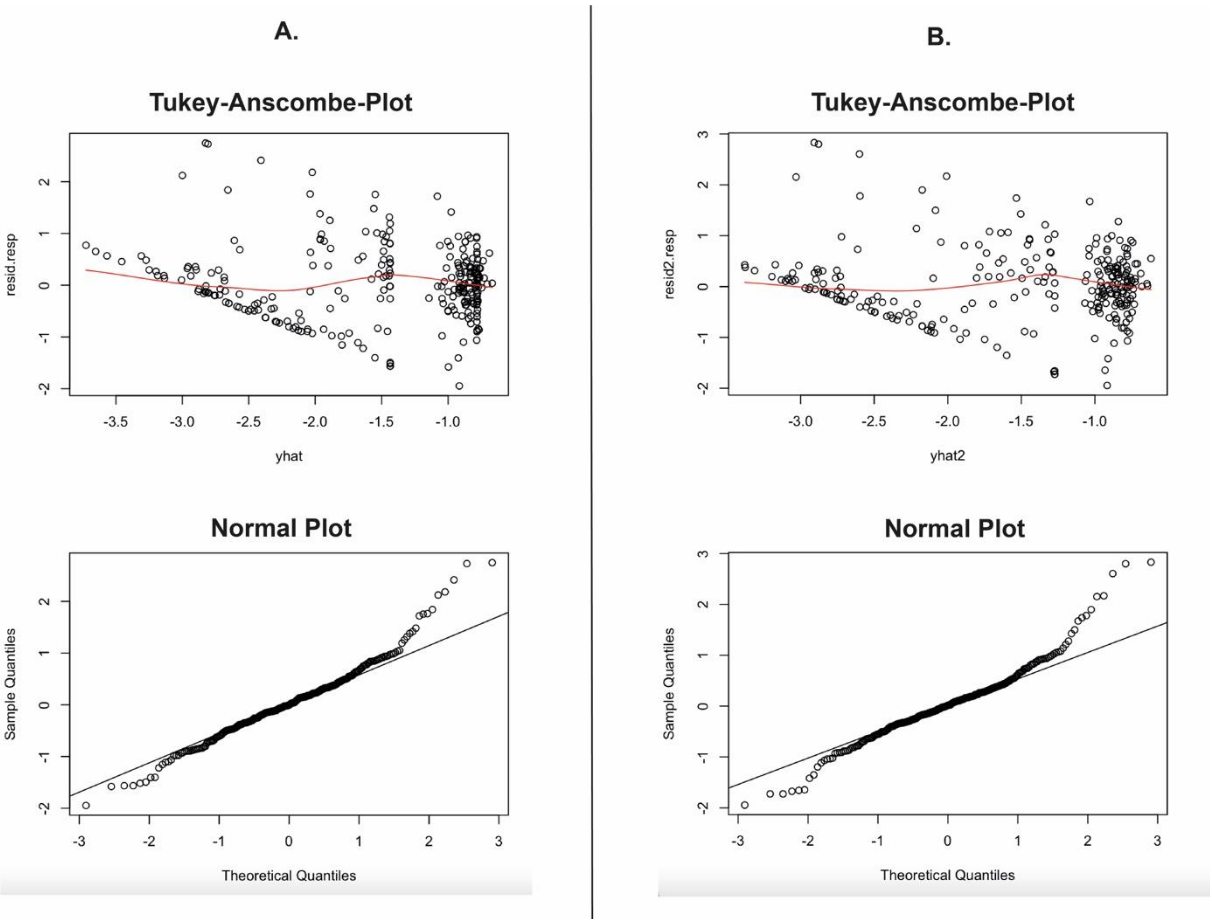

Figure S2: Distribution of the residuals from the dataset resulting from the antiviral screening using the Tobit regression model (*fit.tobit* function). A. Tukey-Anscombe plot and Normal plot generated using the variance of each explanatory variable. **B.** Tukey-Anscombe plot and Normal plot after integrating the square root of each explanatory variable in the model.

In absence of a normal distribution of the residuals, the tobit model was finally integrated with mixed effects, to include additional effects that are not captured by the explanatory variables (e.g., biological variability among replicates). The data were prepared for analysis in the

CensReg library. As indicated in the previous tobit model, the use of the square root improved the quality of the resulting model ($LL_{fit.CensReg}$ = -762.574; $LL_{fit.CensReg2}$= -769.266).

Supplementary data

Protease activity measurement following inhibitor addition in lake water

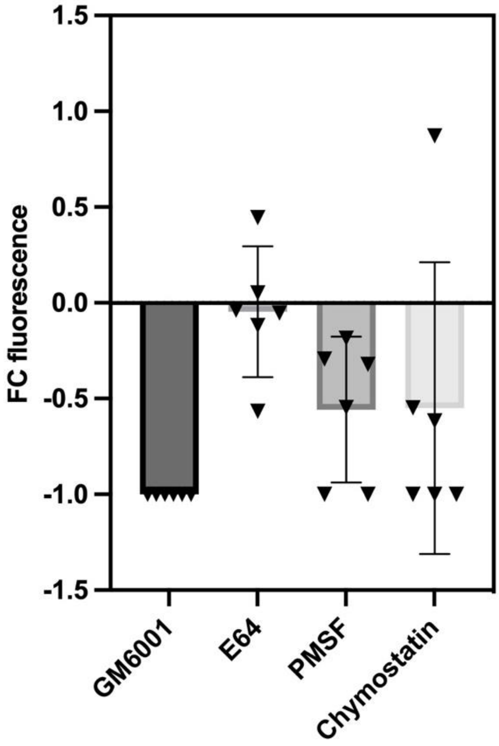

Figure S3 : Fold change in general protease activity between raw and inhibitor-treated lake water samples measured by fluorescence (*pgen*). While the E64 did not reduce the general protease activity of the samples, PMSF, GM6001 and chymostatin induced a total or partial inhibition of general protease activity in lake water samples. Note that E64 exhibited autofluorescence that interfered with *pgen* measurement, such that results for this inhibitor are inconclusive.

1. Ferguson M, Ihrie J. Most probable number and other microbial enumeration techniques. 2019.

2. Altschul SF, Gish W, Miller W, Myers EW, Lipman DJ. Basic local alignment search tool. *J Mol Biol* 1990; 215: 403–410.

3. Yee TW, Wild CJ. Vector generalized additive models. *J Royal Statistical Soc Ser B Methodol* 1996; 58: 481–493.

4. Yee TW. Vector generalized linear and additive models, with an implementation in R. 2015.
