# Supplementary material for: Bacterial matrix metalloproteases and serine proteases contribute to the extra-host inactivation of enteroviruses in lake water
Source: ISME J. 2022 May 11;16(8):1970–9. doi: 10.1038/s41396-022-01246-3 (PMC9296489; doi:10.1038/s41396-022-01246-3)
Supplement: Supplementary file 2 — Supplementary table 1 [file 41396_2022_1246_MOESM2_ESM.pdf]

Sequence_ID	Organism	strain	Accession number
Seq_1	*Achromobacter* sp.	L3768	OK501985
Seq_2	*Acidovorax* sp.	L3002	OK501986
Seq_3	*Acidovorax* sp.	L3770	OK501987
Seq_4	*Acidovorax* sp.	L30180	OK501988
Seq_5	*Acidovorax* sp.	L22109	OK501989
Seq_6	*Acinetobacter* sp.	L3013	OK501990
Seq_7	*Acinetobacter* sp.	L3030	OK501991
Seq_8	*Acinetobacter* sp.	L3031	OK501992
Seq_9	*Acinetobacter* sp.	L3750	OK501993
Seq_10	*Acinetobacter* sp.	L3752	OK501994
Seq_11	*Acinetobacter* sp.	L2284	OK501995
Seq_12	*Acinetobacter* sp.	L22108	OK501996
Seq_13	*Acinetobacter* sp.	L22102	OK501997
Seq_14	*Acinetobacter* sp.	L37140	OK501998
Seq_15	*Aeromonas* sp.	L3003	OK501999
Seq_16	*Aeromonas* sp.	L22130	OK502000
Seq_17	*Aeromonas* sp.	L30160	OK502001
Seq_18	*Aeromonas* sp.	L22126	OK502002
Seq_19	*Aeromonas* sp.	L37149	OK502003
Seq_20	*Aeromonas* sp.	L37135	OK502004
Seq_21	*Aeromonas* sp.	L3017	OK502005
Seq_22	*Aeromonas* sp.	L22119	OK502006
Seq_23	*Aeromonas* sp.	L30168	OK502007
Seq_24	*Aeromonas* sp.	L3758	OK502008
Seq_25	*Aeromonas* sp.	L2280	OK502009
Seq_26	*Aeromonas* sp.	L2282	OK502010
Seq_27	*Aeromonas* sp.	L2283	OK502011
Seq_28	*Aeromonas* sp.	L2292	OK502012
Seq_29	*Arcicella* sp.	L22132	OK502013
Seq_30	*Bacillus* sp.	L37137	OK502014
Seq_31	*Bacillus* sp.	L37150	OK502015
Seq_32	*Bacillus* sp.	L37156	OK502016
Seq_33	*Methylobacterium* sp.	L30183	OK502017
Seq_34	*Brevundimonas* sp.	L3010	OK502018
Seq_35	*Brevundimonas* sp.	L3011	OK502019
Seq_36	*Brevundimonas* sp.	L3012	OK502020
Seq_37	*Brevundimonas* sp.	L3015	OK502021
Seq_38	*Brevundimonas* sp.	L3028	OK502022
Seq_39	*Brevundimonas* sp.	L3032	OK502023
Seq_40	*Brevundimonas* sp.	L3751	OK502024
Seq_41	*Brevundimonas* sp.	L3038	OK502025
Seq_42	*Brevundimonas* sp.	L3746	OK502026
Seq_43	*Brevundimonas* sp.	L3749	OK502027
Seq_44	*Brevundimonas* sp.	L3753	OK502028
Seq_45	*Brevundimonas* sp.	L3756	OK502029
Seq_46	*Brevundimonas* sp.	L3761	OK502030
Seq_47	*Brevundimonas* sp.	L3765	OK502031
Seq_48	*Chryseobacterium* sp.	L3023	OK502032
Seq_49	*Chryseobacterium* sp.	L22104	OK502033
Seq_50	*Citrobacter* sp.	L3041	OK502034
Seq_51	*Citrobacter* sp.	L3042	OK502035
Seq_52	*Cloacibacterium* sp.	L37142	OK502036
Seq_53	*Curvibacter* sp.	L3016	OK502037
Seq_54	*Curvibacter* sp.	L22105	OK502038
Seq_55	*Curvibacter* sp.	L22110	OK502039
Seq_56	*Curvibacter* sp.	L22114	OK502040
Seq_57	*Curvibacter* sp.	L22115	OK502041
Seq_58	*Deinococcus* sp.	L30179	OK502042
Seq_59	*Deinococcus* sp.	L37141	OK502043
Seq_60	*Duganella* sp.	L2275	OK502044
Seq_61	*Duganella* sp.	L22131	OK502045
Seq_62	*Massilia* sp.	L2286	OK502046
Seq_63	*Dyadobacter* sp.	L3021	OK502047
Seq_64	*Dyadobacter* sp.	L3748	OK502048
Seq_65	*Elizabethkingia* sp.	L3027	OK502049
Seq_66	*Buttiauxella* sp.	L2294	OK502050
Seq_67	*Exiguobacterium* sp.	L37145	OK502051
Seq_68	*Flavobacterium* sp.	L2279	OK502052
Seq_69	*Flavobacterium* sp.	L22122	OK502053
Seq_70	*Flavobacterium* sp.	L2281	OK502054
Seq_71	*Flavobacterium* sp.	L2285	OK502055
Seq_72	*Flavobacterium* sp.	L2293	OK502056
Seq_73	*Flavobacterium* sp.	L22113	OK502057

Seq_74	*Hydrogenophaga* sp.	L37136	OK502058
Seq_75	*Janthinobacterium* sp.	L22123	OK502059
Seq_76	*Janthinobacterium* sp.	L22124	OK502060
Seq_77	*Massilia* sp.	L2291	OK502061
Seq_78	*Massilia* sp.	L37133	OK502062
Seq_79	*Massilia* sp.	L22106	OK502063
Seq_80	*Microbacterium* sp.	L3744	OK502064
Seq_81	*Microbacterium* sp.	L3766	OK502065
Seq_82	*Microbacterium* sp.	L3767	OK502066
Seq_83	*Microbacterium* sp.	L3769	OK502067
Seq_84	*Microbacterium* sp.	L3771	OK502068
Seq_85	*Brucella* sp.	L3001	OK502069
Seq_86	*Brucella* sp.	L3005	OK502070
Seq_87	*Brucella* sp.	L3039	OK502071
Seq_88	*Brucella* sp.	L3773	OK502072
Seq_89	*Pseudomonas* sp.	L22107	OK502073
Seq_90	*Pseudomonas* sp.	L22116	OK502074
Seq_91	*Pseudomonas* sp.	L22112	OK502075
Seq_92	*Pseudomonas* sp.	L30161	OK502076
Seq_93	*Pseudomonas* sp.	L30164	OK502077
Seq_94	*Curvibacter* sp.	L22103	OK502078
Seq_95	*Serratia* sp.	L3009	OK502079
Seq_96	*Serratia* sp.	L2296	OK502080
Seq_97	*Sphingomonas* sp.	L30170	OK502081
Seq_98	*Stenotrophomonas* sp.	L3006	OK502082
Seq_99	*Stenotrophomonas* sp.	L30163	OK502083
Seq_100	*Stenotrophomonas* sp.	L3007	OK502084
Seq_101	*Bacillus* sp.	L30177	OK502085
Seq_102	*Stenotrophomonas* sp.	L3018	OK502086
Seq_103	*Stenotrophomonas* sp.	L3019	OK502087
Seq_104	*Stenotrophomonas* sp.	L3020	OK502088
Seq_105	*Stenotrophomonas* sp.	L3022	OK502089
Seq_106	*Stenotrophomonas* sp.	L3024	OK502090
Seq_107	*Stenotrophomonas* sp.	L3025	OK502091
Seq_108	*Stenotrophomonas* sp.	L3026	OK502092
Seq_109	*Stenotrophomonas* sp.	L3029	OK502093
Seq_110	*Stenotrophomonas* sp.	L3033	OK502094
Seq_111	*Stenotrophomonas* sp.	L3035	OK502095
Seq_112	*Stenotrophomonas* sp.	L3036	OK502096
Seq_113	*Stenotrophomonas* sp.	L3037	OK502097
Seq_114	*Stenotrophomonas* sp.	L3040	OK502098
Seq_115	*Stenotrophomonas* sp.	L3743	OK502099
Seq_116	*Stenotrophomonas* sp.	L3745	OK502100
Seq_117	*Stenotrophomonas* sp.	L3747	OK502101
Seq_118	*Stenotrophomonas* sp.	L3754	OK502102
Seq_119	*Stenotrophomonas* sp.	L3755	OK502103
Seq_120	*Stenotrophomonas* sp.	L3757	OK502104
Seq_121	*Stenotrophomonas* sp.	L3759	OK502105
Seq_122	*Stenotrophomonas* sp.	L3760	OK502106
Seq_123	*Stenotrophomonas* sp.	L3762	OK502107
Seq_124	*Stenotrophomonas* sp.	L3763	OK502108
Seq_125	*Stenotrophomonas* sp.	L3772	OK502109
Seq_126	*Stenotrophomonas* sp.	L2277	OK502110
Seq_127	*Stenotrophomonas* sp.	L2278	OK502111
Seq_128	*Stenotrophomonas* sp.	L2295	OK502112
Seq_129	*Stenotrophomonas* sp.	L2297	OK502113
Seq_130	*Stenotrophomonas* sp.	L2298	OK502114
Seq_131	*Stenotrophomonas* sp.	L22100	OK502115
Seq_132	*Streptomyces* sp.	L37147	OK502116
Seq_133	*Streptomyces* sp.	L37146	OK502117
Seq_134	*Pararheinheimera* sp.	L22101	OK502118
Seq_135	*Variovorax* sp.	L3004	OK502119
Seq_136	*Flavobacterium* sp.	L22125	Not submitted (suspected Chimera)

```
>Seq_136_Flavobacterium sp. L22125
ATCTACCTTTTACAGAGGGATAGCCCAGAGAAATTTGGATTAATACCTCATAGTATGCAGAGTTGGCATCAGCACTGCAT
TAAAGTCACAACGGTAAAAGATGAGCATGCGTCCCATTAGCTAGTTGGTAAGGTAACGGCTTACCAAGGCTACGATGGGT
AGGGGTCCTGAGAGGGAGATCCCCCACACTGGTACTGAGACACGGACCAGACTCCTACGGGAGGCAGCAGTGAGGAATAT
TGGACAATGGGCGCAAGCCTGATCCAGCCATGCCGCGTGCAGGATGACGGTCCTATGGATTGTAAACTGCTTTTGCACAG
GAAGAAACAACATTACGTGTAATGTCTTGACGGTACTGTGAGAATAAGGATCGGCTAACTCCGTGCCAGCAGCCGCGGTA
ATACGGAGGATCCAAGCGTTATCCGGAATCATTGGGTTTAAAGGGTCCGTAGGCGGTTTAGTAAGTCAGTGGTGAAAGCC
CATCGCTCAACGGTGGAACGGCCATTGATACTGCTAGACTTGAATTATTAGGAAGTAACTTGAATATGTAGTGTAGCGGT
```
