# Supplementary material for: Bacterial matrix metalloproteases and serine proteases contribute to the extra-host inactivation of enteroviruses in lake water
Source: ISME J. 2022 May 11;16(8):1970–9. doi: 10.1038/s41396-022-01246-3 (PMC9296489; doi:10.1038/s41396-022-01246-3)
Supplement: Supplementary file 3 — Supplementary table 2 [file 41396_2022_1246_MOESM3_ESM.pdf]

										CV-A9 decay (virus 1) Log(Cexp/Cctrl)					Echo-11 decay (virus 2) Log(Cexp/Cctrl)				
Isolates				Protease production on Agar (with bacteria)		Protease act with kit		Id 16S bacteria		triplicate			mean n1-n3		triplicate			mean n1-n3	
Isolate code	Isolate name	Sampling	Isolation temperature	Casein mm (Cas)	Gelatin mm (Gel)	MMP kit (nmol/min/mL)	General kit Pgen (nmol/min/mL)	Genus	closest species	n1	n2	n3	mean	SD	n1	n2	n3	mean	SD
**L22100**	100	nov-19	22	20	12	125	123088	*Stenotrophomonas*	*maltophilia*	-3,000	-2,508	-1,795	-2,434	0,606	-0,572	-0,746	-0,156	-0,491	0,303
**L22101**	101	May-20	22	0	14	7043	15705	*Pararheinheimera*	*tilapiae*	-2,508	-2,860	-3,000	-2,789	0,254	-2,293	-2,034	-1,746	-2,024	0,273
**L22102**	102	May-20	22	0	0	2702	36007	*Acinetobacter*	*guillouiae*	-2,508	-2,860	-3,000	-2,789	0,254	-1,156	-1,156	-0,929	-1,080	0,131
**L22103**	103	May-20	22	0	2	284	133546	*Curvibacter*	*sp.*	-0,646	-0,053	-0,128	-0,276	0,323	-0,899	-0,572	-0,572	-0,681	0,189
**L22104**	104	May-20	22	0	14	5280	55859	*Chryseobacterium*	*treverense*	-3,000	-3,000	-3,000	-3,000	0,000	-2,963	-2,612	-2,156	-2,577	0,405
**L22105**	105	May-20	22	0	0	399	4410	*Curvibacter*	*sp.*	-1,265	-1,265	-1,469	-1,333	0,118	-1,899	-1,572	-1,572	-1,681	0,189
**L22106**	106	May-20	22	13	11	2407	120727	*Massilia*	*aurea*	-3,000	-3,000	-2,860	-2,953	0,081	-1,746	-1,368	-1,156	-1,423	0,299
**L22107**	107	May-20	22	3	8	4474	18865	*Pseudomonas*	*helmanticensis*	-3,000	-3,000	-3,000	-3,000	0,000	-1,156	-0,929	-0,899	-0,995	0,140
**L22108**	108	May-20	22	0	0	2	1911	*Acinetobacter*	*beijerinckii*	-1,469	-1,053	-1,265	-1,262	0,208	-0,746	-0,572	-0,421	-0,580	0,162
**L22109**	109	May-20	22	4	16	1817	25050	*Acidovorax*	*delafieldii*	-0,362	0,205	0,176	0,006	0,319	-0,899	-0,572	-0,572	-0,681	0,189
**L22110**	110	May-20	22	0	0	1	1587	*Curvibacter*	*sp.*	-0,318	0,069	-0,128	-0,126	0,194	-1,156	-0,936	-0,899	-0,997	0,139
**L22112**	112	May-20	22	0	0	40	0	*Pseudomonas*	*peli*	-1,795	-1,265	-1,318	-1,459	0,292	-0,572	-0,572	-0,421	-0,522	0,087
**L22113**	113	May-20	22	6	10	3966	37694	*Flavobacterium*	*spartansii*	-3,000	-2,860	-2,860	-2,907	0,081	-1,156	-1,034	-1,034	-1,074	0,070
**L22114**	114	May-20	22	0	0	1	1587	*Curvibacter*	*sp.*	-1,128	-1,265	-1,053	-1,149	0,108	-0,899	-0,746	-0,572	-0,739	0,164
**L22115**	115	May-20	22	0	0	741	0	*Curvibacter*	*sp.*	-0,795	-1,265	-1,265	-1,108	0,271	-0,899	-0,899	-0,899	-0,899	0,000
**L22116**	116	May-20	22	3	0	5445	1349	*Pseudomonas*	*peli*	-1,265	-1,053	-0,931	-1,083	0,169	-1,156	-1,034	-0,830	-1,006	0,165
**L22119**	119	May-20	22	14	18	1175	169815	*Aeromonas*	*salmonicida*	-3,000	-3,000	-2,860	-2,953	0,081	-2,156	-1,899	-1,899	-1,985	0,148
**L22122**	122	May-20	22	16	8	2262	19852	*Flavobacterium*	*araucananum*	-3,000	-3,000	-3,000	-3,000	0,000	-1,368	-1,232	-0,746	-1,115	0,327
**L22123**	123	May-20	22	2	10	4750	57071	*Janthinobacterium*	*svalbardensis*	-3,000	-3,000	-3,000	-3,000	0,000	-2,660	-2,427	-1,899	-2,329	0,390
**L22124**	124	May-20	22	4	10	4692	57396	*Janthinobacterium*	*svalbardensis*	-3,000	-3,000	-3,000	-3,000	0,000	-2,963	-2,963	-2,660	-2,862	0,175
**L22125**	125	May-20	22	0	0	0	0	*Flavobacterium*	*psychrolimnae*	-1,469	-1,643	-1,265	-1,459	0,189	-0,899	-0,572	-0,572	-0,681	0,189
**L22126**	126	May-20	22	0	16	3431	18166	*Aeromonas*	*media*	-3,000	-3,000	-2,508	-2,836	0,284	-1,746	-1,421	-0,746	-1,304	0,510
**L22130**	130	May-20	22	4	12	202	2749	*Aeromonas*	*rivipollensis*	-3,000	-2,860	-3,000	-2,953	0,081	-1,572	-1,368	-1,368	-1,436	0,118
**L22131**	131	May-20	22	0	10	1650	42766	*Duganella*	*phyllosphaerae*	-3,000	-3,000	-2,860	-2,953	0,081	-0,929	-0,572	-0,572	-0,691	0,206
**L22132**	132	May-20	22	0	0	361	-200	*Arcicella*	*rigui*	-1,265	-1,469	-1,128	-1,287	0,171	-1,572	-1,034	-0,899	-1,168	0,356
**L2275**	75	nov-19	22	0	0	2033	17903	*Duganella*	*phyllosphaerae*	-3,000	-2,860	-2,508	-2,789	0,254	0,101	0,101	0,254	0,152	0,089
**L2277**	77	nov-19	22	20	17	230	128729	*Stenotrophomonas*	*maltophilia*	-3,000	-2,860	-2,053	-2,638	0,511	-0,899	-0,899	-0,746	-0,848	0,089
**L2278**	78	nov-19	22	20	12	720	123882	*Stenotrophomonas*	*maltophilia*	-2,860	-2,508	-2,267	-2,545	0,298	-0,572	-0,746	-0,421	-0,580	0,162
**L2279**	79	nov-19	22	4	8	122	9202	*Flavobacteirum*	*piscis*	-3,000	-3,000	-2,553	-2,851	0,258	-0,368	-0,156	-0,034	-0,186	0,169
**L2280**	80	nov-19	22	24	10	236	113387	*Aeromonas*	*salmonicida*	-3,000	-2,323	-2,267	-2,530	0,408	-0,572	-0,368	-0,368	-0,436	0,118
**L2281**	81	nov-19	22	4	0	457	2392	*Flavobacterium*	*piscis*	-3,000	-3,000	-2,860	-2,953	0,081	-0,899	-0,572	-0,368	-0,613	0,268
**L2282**	82	nov-19	22	0	8	0	9208	*Aeromonas*	*rivipollensis*	-2,860	-3,000	-3,000	-2,953	0,081	-0,899	-0,746	-0,368	-0,671	0,273
**L2283**	83	nov-19	22	20	10	2963	36732	*Aeromonas*	*salmonicida*	-2,860	-2,860	-2,508	-2,743	0,203	-0,899	-0,232	-0,156	-0,429	0,409
**L2284**	84	nov-19	22	0	0	52	0	*Acinetobacter*	*cumulans*	-0,265	-0,265	-0,643	-0,391	0,218	-1,746	-1,572	-1,156	-1,491	0,303
**L2285**	85	nov-19	22	3	4	842	6141	*Flavobacterium*	*piscis*	-3,000	-2,508	-2,508	-2,672	0,284	-0,572	-0,572	-0,368	-0,504	0,118
**L2286**	86	nov-19	22	0	7	6904	30916	*Massilia*	*buxea*	-2,593	-2,898	-2,860	-2,784	0,166	-0,156	-0,034	0,254	0,022	0,210
**L2291**	91	nov-19	22	8	0	842	0	*Massilia*	*aurea*	-0,931	-1,265	-1,053	-1,083	0,169	-0,899	-0,572	-0,368	-0,613	0,268
**L2292**	92	nov-19	22	25	10	5236	31022	*Aeromonas*	*salmonicida*	-3,000	-3,000	-2,860	-2,953	0,081	-0,368	-0,156	0,428	-0,032	0,412
**L2293**	93	nov-19	22	0	0	484	2093	*Flavobacterium*	*piscis*	-0,795	-1,053	-1,128	-0,992	0,175	-0,572	-0,746	-0,034	-0,451	0,371
**L2294**	94	nov-19	22	3	0	0	1936	*Buttiauxella*	*brennerae*	-0,053	-0,643	-0,833	-0,509	0,407	-0,899	-0,746	-0,746	-0,797	0,089
**L2295**	95	nov-19	22	16	11	5459	102786	*Stenotrophomonas*	*maltophilia*	-3,000	-2,860	-2,860	-2,907	0,081	-0,572	-0,368	-0,232	-0,391	0,171
**L2296**	96	nov-19	22	0	0	4177	37456	*Serratia*	*quinivorans*	-3,000	-2,860	-2,508	-2,789	0,254	0,101	-0,156	0,428	0,124	0,293
**L2297**	97	nov-19	22	18	17	315	119378	*Stenotrophomonas*	*maltophilia*	-2,267	-2,860	-2,508	-2,545	0,298	-1,156	-1,156	-0,746	-1,019	0,237
**L2298**	98	nov-19	22	19	14	283	126861	*Stenotrophomonas*	*maltophilia*	-2,860	-3,000	-3,000	-2,953	0,081	-0,899	-0,368	-0,156	-0,474	0,383
**L3001**	1	nov-19	30	0	0	1424	0	*Ochrobactrum*	*rhizospherae*	-0,053	-0,128	-0,053	-0,078	0,044	-1,368	-0,746	-0,368	-0,828	0,505
**L3002**	2	nov-19	30	0	0	5	0	*Acidovorax*	*delafieldii*	-0,931	-1,053	-1,053	-1,012	0,070	-0,232	-0,156	-0,034	-0,141	0,100
**L3003**	3	nov-19	30	14	18	1789	3311	*Aeromonas*	*salmonicida*	-3,000	-3,000	-3,000	-3,000	0,000	-1,156	-1,156	-0,572	-0,961	0,337
**L3004**	4	nov-19	30	0	20	351	144503	*Variovorax*	*paradoxus*	-3,000	-3,000	-2,508	-2,836	0,284	0,254	0,428	0,632	0,438	0,189
**L3005**	5	nov-19	30	0	0	38	0	*Ochrobactrum*	*rhizospherae*	-0,931	-0,931	-0,931	-0,931	0,000	-0,746	-0,368	-0,034	-0,383	0,356
**L3006**	6	nov-19	30	8	20	244	232577	*Stenotrophomonas*	*maltophilia*	-3,000	-2,860	-2,508	-2,789	0,254	-1,572	-1,156	-0,899	-1,209	0,339
**L3007**	7	nov-19	30	8	16	8702	89443	*Stenotrophomonas*	*maltophilia*	-2,860	-2,508	-2,860	-2,743	0,203	-1,746	-1,572	-1,156	-1,491	0,303
**L3009**	9	nov-19	30	11	14	9459	149937	*Serratia*	*quinivorans*	-3,000	-3,000	-3,000	-3,000	0,000	-0,572	-0,572	-0,572	-0,572	0,000
**L3010**	10	nov-19	30	0	0	5737	0	*Brevundimonas*	*mediterraneae*	-1,469	-0,643	-1,128	-1,080	0,415	-1,572	-1,572	-1,572	-1,572	0,000
**L3011**	11	nov-19	30	0	0	5942	837	*Brevundimonas*	*bullata*	-0,795	-1,053	-1,053	-0,967	0,149	-0,572	-0,572	-0,572	-0,572	0,000
**L3012**	12	nov-19	30	0	5	2751	1487	*Brevundimonas*	*mediterraneae*	-0,939	-1,318	-1,265	-1,174	0,205	-0,572	-0,572	-0,368	-0,504	0,118
**L3013**	13	nov-19	30	0	0	4	0	*Acinetobacter*	*johnsonii*	-0,795	-1,265	-0,931	-0,997	0,242	-1,572	-1,368	-0,368	-1,103	0,644
**L3015**	15	nov-19	30	0	12	146	120077	*Brevundimonas*	*mediterraneae*	-2,508	-2,508	-3,000	-2,672	0,284	-0,572	-0,156	0,632	-0,032	0,611
**L3016**	16	nov-19	30	0	0	219	2823	*Curvibacter*	*sp.*	-0,469	-0,643	-0,469	-0,527	0,101	-0,936	-0,746	-0,572	-0,751	0,182
**L30160**	160	May-20	30	5	0	122	0	*Aeromonas*	*rivipollensis*	-2,553	-2,267	-2,267	-2,363	0,165	-1,746	-1,572	-1,572	-1,630	0,100
**L30161**	161	May-20	30	10	0	219	5110	*Pseudomonas*	*monteilli*	-2,860	-2,860	-2,860	-2,860	0,000	-0,746	-0,746	-0,899	-0,797	0,088
**L30163**	163	May-20	30	23	11	2523	27599	*Stenotrophomonas*	*rhizophila*	-3,000	-3,000	-3,000	-3,000	0,000	-1,035	-1,156	-1,369	-1,186	0,169
**L30164**	164	May-20	30	0	0	276	17179	*Pseudomonas*	*monteilli*	-2,860	-0,795	-0,265	-1,307	1,371	-0,746	-0,422	-0,746	-0,638	0,188
**L30168**	168	May-20	30	6	15	572	2711	*Aeromonas*	*rivipollensis*	-3,000	-2,860	-2,860	-2,907	0,081	-1,572	-1,572	-1,369	-1,504	0,118
**L3017**	17	nov-19	30	0	0	0	64505	*Aeromonas*	*rivipollensis*	-0,931	-1,318	-1,318	-1,189	0,223	-0,830	-0,572	-0,368	-0,590	0,231
**L30170**	170	May-20	30	0	0	0	0	*Sphingomonas*	*koreensis*	-3,000	-3,000	-3,000	-3,000	0,000	-1,035	-1,369	-1,572	-1,325	0,271
**L30177**	177	May-20	30	0	0	0	0	*Bacillus*	*mojavensis*	-1,128	-1,469	-1,017	-1,205	0,235	-0,746	-0,605	-0,572	-0,641	0,092
**L30179**	179	May-20	30	0	0	127	2461	*Deinococcus*	*aquaticus*	-2,508	-2,267	-2,267	-2,348	0,139	-1,572	-1,572	-1,035	-1,393	0,310
**L3018**	18	nov-19	30	16	12	5385	39830	*Stenotrophomonas*	*maltophilia*	-0,053	-0,128	-0,053	-0,078	0,044	-0,746	-0,572	-0,156	-0,491	0,303
**L30180**	180	May-20	30	25	18	306	11457	*Acidovorax*	*facilis*	-0,833	-0,824	-0,795	-0,817	0,020	-1,369	-1,746	-1,035	-1,383	0,356
**L30183**	183	May-20	30	0	0	0	0	*Methylobacterium*	*organophilum*	-0,736	-0,531	0,531	-0,245	0,680	-0,736	-1,035	-1,369	-1,046	0,316
**L3019**	19	nov-19	30	15	11	1261	126637	*Stenotrophomonas*	*maltophilia*	-2,860	-3,000	-2,508	-2,789	0,253	-1,572	-1,034	-0,899	-1,168	0,356
**L3020**	20	nov-19	30	15	19	278	109920	*Stenotrophomonas*	*maltophilia*	-3,000	-3,000	-1,795	-2,598	0,696	-1,746	-1,572	-1,232	-1,517	0,261
**L3021**	21	nov-19	30	0	0	982	14917	*Dyadobacter*	*endophyticus*	-1,469	-1,643	-1,265	-1,459	0,189	-1,368	-1,232	-1,156	-1,252	0,108
**L3022**	22	nov-19	30	14	16	0	0	*Stenotrophomonas*	*maltophilia*	-1,643	-3,000	-3,000	-2,548	0,784	-0,572	-0,368	-0,368	-0,436	0,118
**L3023**	23	nov-19	30	6	20	2862	60007	*Chryseobacterium*	*carnipullorum*	-0,053	-0,053	-0,128	-0,078	0,044	-1,156	-1,156	-0,899	-1,070	0,148
**L3024**	24	nov-19	30	14	10	6089	40017	*Stenotrophomonas*	*maltophilia*	-2,860	-3,000	-3,000	-2,953	0,081	-0,899	-0,746	-0,572	-0,739	0,164
**L3025**	25	nov-19	30	14	20	234	199638	*Stenotrophomonas*	*maltophilia*	-2,860	-3,000	-3,000	-2,953	0,081	-1,156	-1,156	-0,746	-1,019	0,237
**L3026**	26	nov-19	30	14	16	10630	129685	*Stenotrophomonas*	*maltophilia*	-3,000	-2,860	-3,000	-2,953	0,081	-1,368	-1,156	-1,034	-1,186	0,169
**L3027**	27	nov-19	30	15	20	6276	40542	*Elizabethkingia*	*miricola*	-3,000	-2,860	-3,000	-2,930	0,099	-0,572	-0,156	-0,156	-0,294	0,240
**L3028**	28	nov-19	30	0	0	0	30260	*Brevundimonas*	*diminuta*	0,205	0,205	0,205	0,205	0,000	-0,572	-0,572	-0,156	-0,433	0,240
**L3029**	29	nov-19	30	11	11	339	18078	*Stenotrophomonas*	*maltophilia*	-3,000	-3,000	-3,000	-3,000	0,000	-1,368	-1,156	-0,572	-1,032	0,412
**L3030**	30	nov-19	30	0	0	26	0	*Acinetobacter*	*johnsonii*	-0,441	-0,795	-0,643	-0,626	0,178	-1,572	-1,368	0,071	-0,956	0,896
**L3031**	31	nov-19	30	0	0	18	0	*Acinetobacter*	*johnsonii*	-0,643	-0,795	-0,469	-0,636	0,163	-1,368	-1,156	-0,929	-1,151	0,220
**L3032**	32	nov-19	30	0	0	5247	0	*Brevundimonas*	*bullata*	-0,643	-0,795	-0,469	-0,636	0,163	-1,368	-1,156	-0,368	-0,964	0,527
**L3033**	33	nov-19	30	13	22	7318	125475	*Stenotrophomonas*	*maltophilia*	-3,000	-3,000	-3,000	-3,000	0,000	-1,572	-1,368	-1,232	-1,391	0,171
**L3035**	35	nov-19	30	12	20	227	124475	*Stenotrophomonas*	*maltophilia*	-3,000	-3,000	-2,267	-2,756	0,423	-1,232	-1,034	-0,840	-1,035	0,196
**L3036**	36	nov-19	30	11	16	15	119665	*Stenotrophomonas*	*maltophilia*	-3,000	-2,860	-2,860	-2,907	0,081	-1,746	-1,572	-1,156	-1,491	0,303
**L3037**	37	nov-19	30	9	16	1996	114443	*Stenotrophomonas*	*maltophilia*	-3,000	-3,000	-3,000	-3,000	0,000	-0,899	-0,572	-0,156	-0,542	0,373
**L3038**	38	nov-19	30	0	0	5396	0	*Brevundimonas*	*bullata*	-1,469	-1,643	-1,469	-1,527	0,101	-0,746	-0,572	-0,156	-0,491	0,303
**L3039**	39	nov-19	30	0	0	1	0	Ochrobactrum	rhizospherae	-3,000	-2,860	-3,000	-2,953	0,081	-0,899	-0,746	-0,746	-0,797	0,089
**L3040**	40	nov-19	30	11	16	2029	116129	*Stenotrophomonas*	*maltophilia*	-3,000	-3,000	-3,000	-3,000	0,000	-1,232	-0,899	-0,746	-0,959	0,249
**L3041**	41	nov-19	30	14	16	51	129173	*Citrobacter*	*freundii*	-3,000	-2,860	-3,000	-2,953	0,081	-1,746	-0,746	-0,572	-1,021	0,634
**L3042**	42	nov-19	30	3	0	8	0	*Citrobacter*	*freundii*	-0,795	-1,053	-0,643	-0,830	0,207	-1,899	-1,572	-1,368	-1,613	0,268
**L37133**	133	May-20	37	3	0	2563	69215	*Massilia*	*niastensis*	-0,643	-0,643	-0,469	-0,585	0,101	-0,669	-0,131	0,004	-0,265	0,356
**L37135**	135	May-20	37	4	20	57	1074	*Aeromonas*	*hydrophila*	-3,000	-2,860	-2,508	-2,789	0,254	0,234	0,772	0,907	0,638	0,356

**L37136**	136	May-20	37	0	0	0	0	*Hydrogenophaga*	*taeniospiralis*	-0,643	0,531	-1,053	-2,930	0,830	-2,034	-1,572	-1,368	-1,658	0,341
**L37137**	137	May-20	37	8	20	2487	106747	*Bacillus*	*stratosphericus*	-0,643	-1,053	-0,939	-0,878	0,212	-1,131	-0,669	-1,572	-1,124	0,452
**L37140**	140	May-20	37	0	2	5518	17279	*Acinetobacter*	*beijerinckii*	-3,000	-3,000	-2,860	-2,953	0,081	-0,746	-0,572	-1,035	-0,784	0,233
**L37141**	141	May-20	37	3	8	3181	9058	*Deinococcus*	*aquaticus*	-2,860	-2,860	-2,267	-2,662	0,342	-1,035	-0,422	-1,156	-0,871	0,394
**L37142**	142	May-20	37	0	0	470	1886	*Cloacibacterium*	*rupense*	-1,795	-1,469	-1,469	-1,577	0,189	-1,899	-1,746	-1,899	-1,848	0,089
**L37145**	145	May-20	37	18	8	390	2861	*Exiguobacterium*	*undae*	-3,000	-2,860	-2,508	-2,789	0,253	-1,369	-1,369	-1,369	-1,369	0,000
**L37146**	146	May-20	37	4	8	1385	3561	*Streptomyces*	*cellulosae*	-1,643	-0,643	-0,931	-1,072	0,515	-0,572	-1,369	-0,928	-0,956	0,399
**L37147**	147	May-20	37	3	6	876	4985	*Streptomyces*	*cellulosae*	-1,931	-1,824	-2,053	-1,936	0,114	-1,035	-1,156	-1,572	-1,254	0,282
**L37149**	149	May-20	37	4	24	0	0	*Aeromonas*	*rivipollensis*	-3,000	-3,000	-2,860	-2,953	0,081	-1,572	-1,572	-1,035	-1,393	0,310
**L37150**	150	May-20	37	4	20	311	6484	*Bacillus*	*subtilis*	-3,000	-3,000	-3,000	-3,000	0,000	-1,035	-1,369	-0,899	-1,101	0,242
**L37156**	156	May-20	37	14	20	699	244540	Bacillus	subtilis	-3,000	-3,000	-3,000	-3,000	0,000	-1,035	-0,899	-1,572	-1,169	0,356
**L3743**	43	nov-19	37	9	8	6882	98838	*Stenotrophomonas*	*maltophilia*	-3,000	-2,860	-3,000	-2,953	0,081	-0,156	0,071	0,428	0,115	0,294
**L3744**	44	nov-19	37	0	12	311	113412	*Microbacterium*	*maritypicum*	-3,000	-3,000	-3,000	-3,000	0,000	-0,572	-0,368	-0,034	-0,325	0,272
**L3745**	45	nov-19	37	12	12	253	123900	*Stenotrophomonas*	*maltophilia*	-1,795	-1,795	-1,643	-1,744	0,088	-0,899	-0,746	-0,368	-0,671	0,273
**L3746**	46	nov-19	37	0	0	3921	568	*Brevundimonas*	*bullata*	-1,053	-3,000	-2,053	-2,035	0,974	-0,368	-0,368	-0,156	-0,297	0,123
**L3747**	47	nov-19	37	11	11	0	64880	*Stenotrophomonas*	*maltophilia*	-3,000	-3,000	-2,267	-2,756	0,423	-1,746	-1,232	-0,746	-1,241	0,500
**L3748**	48	nov-19	37	0	0	295	114661	*Dyadobacter*	*endophyticus*	-3,000	-2,508	-1,502	-2,337	0,764	0,101	-0,156	-0,156	-0,070	0,148
**L3749**	49	nov-19	37	0	0	371	11019	*Brevundimonas*	*vesicularis*	-2,508	-1,469	-1,318	-1,765	0,648	-1,232	-1,156	-0,369	-0,919	0,478
**L3750**	50	nov-19	37	0	0	6709	387	*Acinetobacter*	*soli*	-3,000	-1,643	-0,265	-1,636	1,368	-0,746	-0,746	-0,368	-0,620	0,218
**L3751**	51	nov-19	37	0	0	15	11475	*Brevundimonas*	*diminuta*	-0,795	-0,643	-0,469	-0,636	0,163	-0,368	-0,605	-0,746	-0,573	0,191
**L3752**	52	nov-19	37	0	0	1959	4004	*Acinetobacter*	*johnsonii*	-0,643	-0,643	-0,469	-0,585	0,101	-0,746	-0,572	-0,368	-0,562	0,189
**L3753**	53	nov-19	37	0	0	664	11357	*Brevundimonas*	*mediterraneae*	-0,795	-0,469	-0,318	-0,527	0,244	-0,605	-0,746	-0,572	-0,641	0,092
**L3754**	54	nov-19	37	11	14	1139	0	*Stenotrophomonas*	*maltophilia*	-3,000	-3,000	-3,000	-3,000	0,000	-1,156	-0,746	-0,572	-0,824	0,300
**L3755**	55	nov-19	37	13	12	0	114243	*Stenotrophomonas*	*maltophilia*	-2,053	-1,795	-1,795	-1,881	0,149	-0,252	0,157	0,331	0,079	0,300
**L3756**	56	nov-19	37	0	0	6909	1174	*Brevundimonas*	*mediterraneae*	-2,053	-2,268	-2,268	-2,160	0,152	-0,899	-0,746	-0,746	-0,797	0,089
**L3757**	57	nov-19	37	14	11	5312	101743	*Stenotrophomonas*	*maltophilia*	-3,000	-3,000	-1,795	-2,598	0,696	-0,899	-0,572	-0,368	-0,613	0,268
**L3758**	58	nov-19	37	11	20	287	91004	*Aeromonas*	*salmonicida*	-3,000	-3,000	-2,860	-2,953	0,081	-0,368	-0,156	-0,156	-0,226	0,123
**L3759**	59	nov-19	37	11	12	7012	112319	*Stenotrophomonas*	*maltophilia*	-3,000	-3,000	-3,000	-3,000	0,000	-1,034	-0,899	-0,746	-0,893	0,144
**L3760**	60	nov-19	37	13	12	7077	115842	*Stenotrophomonas*	*maltophilia*	-3,000	-3,000	-3,000	-3,000	0,000	-1,156	-1,156	-0,368	-0,893	0,455
**L3761**	61	nov-19	37	0	13	371	123226	*Brevundimonas*	*diminuta*	-2,860	-3,000	-3,000	-2,953	0,081	-1,232	-1,034	-0,572	-0,946	0,339
**L3762**	62	nov-19	37	13	13	294	118678	*Stenotrophomonas*	*maltophilia*	-3,000	-3,000	-3,000	-3,000	0,000	-1,746	-1,572	-1,156	-1,491	0,303
**L3763**	63	nov-19	37	13	12	297	129485	*Stenotrophomonas*	*maltophilia*	-3,000	-3,000	-3,000	-3,000	0,000	-1,572	-1,232	-0,572	-1,125	0,508
**L3765**	65	nov-19	37	0	0	6935	0	*Brevundimonas*	*bullata*	-1,469	-1,265	-1,469	-1,401	0,118	-0,899	-0,899	-0,899	-0,899	0,000
**L3766**	66	nov-19	37	0	0	6491	53923	*Microbacterium*	*paraoxydans*	-3,000	-3,000	-3,000	-3,000	0,000	-1,034	-0,746	-0,572	-0,784	0,233
**L3767**	67	nov-19	37	0	0	5426	59795	*Microbacterium*	*paraoxydans*	-3,000	-3,000	-3,000	-3,000	0,000	-0,572	-0,368	0,101	-0,280	0,345
**L3768**	68	nov-19	37	0	14	149	0	*Achromobacter*	*xylosoxidans*	0,069	0,205	0,205	0,160	0,078	-0,156	-0,156	-0,156	-0,156	0,000
**L3769**	69	nov-19	37	4	0	842	129685	*Microbacterium*	*paraoxydans*	-3,000	-2,860	-2,860	-2,907	0,081	-0,746	-0,572	-0,572	-0,630	0,100
**L3770**	70	nov-19	37	0	0	0	0	*Acidovorax*	*temperans*	-1,824	-1,469	-1,795	-1,696	0,197	-0,572	0,254	-0,232	-0,183	0,415
**L3771**	71	nov-19	37	2	0	5222	49688	*Microbacterium*	*saperdae*	-3,000	-3,000	-3,000	-3,000	0,000	-0,746	-0,572	-0,572	-0,630	0,100
**L3772**	72	nov-19	37	12	12	253	143803	*Stenotrophomonas*	*maltophilia*	-3,000	-3,000	-1,795	-2,598	0,696	-1,156	-1,156	-1,156	-1,156	0,000
**L3773**	73	nov-19	37	0	0	0	0	*Ochrobactrum*	*anthropi*	-3,000	-3,000	-3,000	-3,000	0,000	-0,035	-0,035	-0,035	-0,035	0,000
